# Machine learning models for identifying prediabetes: a systematic review of performance, validation, and implications for primary care

**DOI:** 10.3389/fmed.2026.1956800

**Published:** 2026-09-28

**Authors:** Liang Wang, Yan Zhang, Hao Yang, Xiwei Wang, Xinyuan Qi, Li Gou

**Affiliations:** 1 Department of Geriatric Endocrinology, Sichuan Provincial People’s Hospital, University of Electronic Science and Technology of China, Chengdu, China; 2 Department of Medical Affairs, Sichuan Provincial People’s Hospital, University of Electronic Science and Technology of China, Chengdu, China; 3 Department of Gastroenterology, Sichuan Provincial People’s Hospital, University of Electronic Science and Technology of China, Chengdu, China; 4 School of Medicine, University of Electronic Science and Technology of China, Chengdu, China; 5 Department of Nursing Research Center, Sichuan Provincial People’s Hospital, University of Electronic Science and Technology of China, Chengdu, China

**Keywords:** machine learning, prediabetes, prediction model, primary care, screening, systematic review

## Abstract

**Background:**

Machine learning models have been proposed for identifying prediabetes, but their methodological quality, generalisability, and readiness for primary care implementation remain uncertain.

**Methods:**

We conducted a Preferred Reporting Items for Systematic Reviews and Meta-Analyses (PRISMA) 2020-compliant systematic review registered in PROSPERO (CRD420251029165). Eight databases were searched from inception to 1 April 2025, with PubMed, Embase, and Web of Science Core Collection updated through 1 August 2026. The primary review question focused on current/prevalent prediabetes identification; longitudinal/prognostic studies addressing future dysglycaemia were retained only as supplementary evidence and interpreted separately. Principal performance and implementation conclusions were restricted to current/prevalent identification. Two reviewers independently screened studies and extracted data. Risk of bias and applicability were assessed using Prediction Model Risk of Bias Assessment Tool (PROBAST). Findings were synthesised descriptively because of heterogeneity.

**Results:**

Twenty-six studies involving 435,647 participants were included. Of these, 23 studies constituted the primary evidence base for current/prevalent prediabetes identification, whereas three longitudinal/prognostic studies were retained as supplementary evidence and interpreted separately. Across all included evidence, 110 models were reported. Development or internal validation area under the receiver operating characteristic curves (AUCs) ranged from 0.49 to 0.9989 across heterogeneous modelling tasks; the highest value represented overall multiclass discrimination rather than a prediabetes-specific binary AUC. Independent external validation was reported in only one study (1/26, 3.8%), in which the area under the receiver operating characteristic curve (AUROC) for prediabetes decreased from 0.746 in internal evaluation to 0.590 and 0.523 in two external cohorts. Fourteen studies were judged to have a low overall risk of bias, whereas 12 studies (46.2%) were judged to have a high overall risk of bias. Calibration, clinical utility assessment, and subgroup fairness evaluation remained limited.

**Conclusion:**

Machine learning models show potential to support current/prevalent prediabetes identification in primary care, particularly when based on routinely collected variables. However, strong performance observed during model development or internal validation should not be interpreted as evidence of implementation readiness. Given the limited independent external validation and insufficient evidence regarding calibration, clinical utility, fairness, and real-world workflow integration, current evidence remains insufficient to support routine primary care implementation. These conclusions relate to current/prevalent prediabetes identification and should not be extrapolated to longitudinal/prognostic prediction of future prediabetes, impaired fasting glucose, or related dysglycaemic progression.

**Systematic Review Registration:**

PROSPERO CRD420251029165.

## 1 Introduction

Prediabetes, which includes impaired fasting glucose, impaired glucose tolerance, or both, represents an intermediate state between normoglycaemia and diabetes (1, 2). Global estimates indicate a large and growing population with intermediate hyperglycaemia (3), while national survey data demonstrate a substantial burden in China (4). Prediabetes is associated with progression to type 2 diabetes and increased risks of cardiovascular disease and mortality (5, 6). Early identification is clinically important because lifestyle interventions and metformin can delay or prevent diabetes in selected high-risk populations and may be cost-effective (7–9).

Primary care is well positioned to identify individuals at risk, arrange confirmatory testing, initiate lifestyle counselling, and provide longitudinal follow-up. Demographic, anthropometric, biochemical, and lifestyle information is frequently available during routine consultations, community screening, and health examinations. A prediction model that reliably combines these data could help clinicians prioritise testing and follow-up; however, a model that performs well only in its development dataset may increase unnecessary testing or miss high-risk individuals when transferred to a different population.

Machine learning (ML) methods can identify complex and potentially non-linear relationships within electronic health records, health examination datasets, and other clinical data sources (10–12). Recent studies have applied regression, tree-based ensembles, support vector machines, and neural networks to prediabetes identification and related prediction model development (13, 14). For primary care decision support, discrimination alone is insufficient. Models should also be reproducible, calibrated, externally validated, interpretable, and feasible to integrate into clinical workflows (11).

An earlier systematic review included five studies and highlighted heterogeneity and validation-related limitations in the available evidence (15). The evidence base has since expanded, but uncertainty remains regarding predictor selection, validation practices, risk of bias, applicability, and readiness for clinical decision support. It is important to distinguish between machine learning models developed for current/prevalent prediabetes identification and those developed for longitudinal/prognostic prediction of future dysglycaemia. Current/prevalent identification models aim to determine whether prediabetes is present at the time of assessment, whereas longitudinal/prognostic models estimate the probability of developing future prediabetes, impaired fasting glucose, or related dysglycaemic outcomes over a defined follow-up period. These two modelling tasks address fundamentally different clinical questions and therefore require different temporal structures, outcome definitions, validation strategies, and clinical interpretation. Accordingly, this review focused primarily on machine learning models developed for current/prevalent prediabetes identification in primary care settings. In particular, we evaluated their performance, predictor selection, validation practices, methodological quality, and implications for primary care implementation.

## 2 Methods

### 2.1 Study design and protocol

This systematic review followed the Preferred Reporting Items for Systematic Reviews and Meta-Analyses (PRISMA) 2020 statement (16) and was prospectively registered in PROSPERO (CRD420251029165). The completed PRISMA checklist is provided as a separate reporting-guideline file.

### 2.2 Review question and eligibility criteria

The review question was framed using an adapted PICOS structure appropriate for prediction model research. The population comprised children or adults assessed for current/prevalent prediabetes status. The index approaches were ML or statistical learning models developed for identifying current/prevalent prediabetes status. Comparators were alternative models or conventional screening approaches when reported. Outcomes included discrimination and classification performance, such as area under the receiver operating characteristic curve (AUC), sensitivity, specificity, and accuracy. Eligible study designs included model development and/or validation studies using cohort, retrospective, cross-sectional, prospective, or case–control data.

Studies were included when they developed or validated machine learning models for current/prevalent prediabetes identification or related longitudinal/prognostic prediction and reported at least one model performance measure. The adapted PICOS defined current/prevalent prediabetes identification as the primary review question. Longitudinal/prognostic studies addressing future prediabetes, impaired fasting glucose, or related dysglycaemic progression were retained only as supplementary evidence, explicitly identified as such, and interpreted separately from current/prevalent identification models. They were not used to support conclusions regarding the performance, transportability, or implementation readiness of current/prevalent prediabetes identification models. Conference publications were considered eligible only when sufficient methodological details and model performance information were available. In particular, eligible conference records had to provide a clearly defined study population and prediabetes outcome, describe the machine learning algorithm(s), report at least one quantitative performance measure, and provide sufficient information regarding predictors, model development, or validation procedures. Conference abstracts or records lacking adequate information for evaluating model performance or methodological quality were excluded. Publications in English or Chinese were eligible. Reviews, meta-analyses, editorials, case reports, conference abstracts without sufficient data, studies limited to risk factor analysis without model development, and studies without a clearly described modelling process or performance result were excluded. Studies predicting progression from established prediabetes to diabetes were excluded unless current/prevalent prediabetes identification or longitudinal/prognostic prediction of future prediabetes could be evaluated separately.

### 2.3 Information sources and search strategy

PubMed, Embase, Web of Science Core Collection, the Cochrane Library, China National Knowledge Infrastructure, Wanfang, VIP, and the Chinese Biomedical Literature Database were searched from database inception to 1 April 2025. Controlled vocabulary and free-text terms covered machine learning, artificial intelligence, prediction modelling, risk assessment, and prediabetes. In response to peer review, an updated search of PubMed, Embase, and Web of Science Core Collection was conducted from 2 April 2025 to 1 August 2026 using the same database-specific search strategies as in the original search, with only the date limits extended. The same eligibility criteria were applied to records identified through the updated search. Reference lists of eligible articles were also screened. The complete database-specific search strategies, including the updated searches, are provided in Supplementary Material 1.

### 2.4 Study selection and data extraction

Two reviewers independently screened titles and abstracts against the eligibility criteria and then assessed potentially eligible full texts. Disagreements were resolved by discussion or consultation with a third reviewer. Reasons for exclusion at the full-text stage were recorded.

Two reviewers independently extracted study location, design, sample size, population characteristics, outcome definition, candidate and retained predictors, feature selection method, modelling algorithm, missing data handling (and, where reported, its timing relative to model development and validation), validation method, model performance, calibration assessment, and clinical utility assessment (including decision curve analysis and net benefit where reported). Data extraction was informed by the Checklist for Critical Appraisal and Data Extraction for Systematic Reviews of Prediction Modelling Studies (CHARMS) (17). For the purposes of this review, independent external validation was defined as evaluation of an already developed model in an independent participant dataset that was not used for model development, feature selection, hyperparameter optimisation, or internal validation. Random train–test splitting, k-fold cross-validation, nested cross-validation, and holdout subsets derived from the same source dataset were classified as internal validation rather than independent external validation. Extracted data were compared, and discrepancies were resolved by consensus. Study-level missing data handling and its reported relationship to model development and validation are summarised in Supplementary Table S7. Information regarding subgroup performance, fairness assessment, and model evaluation across demographic or clinical subgroups was also extracted when available. During the revision, we additionally extracted the reference standard used to define prediabetes, whether any glycaemic measure contributing directly or indirectly to that reference standard was also used as a model predictor, the temporal relationship between predictor measurement and outcome ascertainment, and whether paired models with and without potentially overlapping glycaemic predictors were reported.

During the peer review revision, ChatGPT (OpenAI; GPT-5.6 Sol) was used only as an assistive tool. ChatGPT did not independently search bibliographic databases or retrieve original study full texts. The inputs provided to ChatGPT consisted of study information previously extracted by the authors and manuscript or supplementary material text supplied for organisation, consistency checking, and language-related assistance. ChatGPT did not make study eligibility decisions, data extraction decisions, Prediction Model Risk of Bias Assessment Tool (PROBAST) judgements, or other risk-of-bias assessments. All literature screening, eligibility decisions, data extraction, risk-of-bias assessments, statistical summaries, and scientific interpretations were performed and manually verified by the authors against the original publications. All AI-assisted outputs were reviewed by the authors before incorporation into the manuscript. The requests/prompts used for AI-assisted tasks are provided in Supplementary Material 3.

### 2.5 Risk of bias and applicability

Risk of bias and applicability were assessed using the PROBAST (18). PROBAST was selected because the included studies were prediction model development and validation studies. It provides a structured framework for evaluating potential bias and applicability concerns in prediction models across predefined domains and has been widely applied in systematic reviews of prediction models. Risk of bias was evaluated across four domains (participants, predictors, outcome, and analysis), whereas applicability concerns were assessed across three domains (participants, predictors, and outcome). Overall judgements followed PROBAST guidance. A high-risk judgement in any risk-of-bias domain resulted in an overall high-risk assessment. Applicability was judged according to the degree of concern that the study population, predictors, or outcome differed from the review question.

Because conventional PROBAST does not explicitly capture all methodological risks specific to machine learning workflows, we additionally performed a structured ML-specific methodological audit for each included study, informed by principles incorporated into PROBAST + AI (19). The reported methods, model development workflow, figures, and supplementary materials were manually reviewed to determine (1) whether training, validation, and test data were appropriately separated; (2) whether imputation, scaling, feature engineering, and feature selection were restricted to the training data or training folds; (3) whether class imbalance procedures were performed only within the training data; (4) whether hyperparameter optimisation and model selection were independent of final performance evaluation; (5) whether nested cross-validation or an equivalent separation procedure was used when cross-validation was used for tuning or model selection; and (6) whether repeated model selection or full-dataset pre-processing could have introduced information leakage or optimistic performance estimates. When reporting was insufficient to determine the procedure, the corresponding item was classified as unclear. Findings from this ML-specific audit were incorporated into the PROBAST analysis domain judgement, with clear evidence of leakage, pre-processing or feature selection outside the validation framework, or tuning/model selection involving evaluation data considered high-risk concerns. The study-level findings of this ML-specific methodological audit are systematically reported in Supplementary Table S5.

Potential incorporation bias was additionally examined on a study-by-study basis. For current/prevalent identification models, use of the same glycaemic measurement as both a model predictor and a component of the reference standard used to define prediabetes was considered a high-risk concern due to predictor–outcome incorporation and was reflected in the relevant PROBAST predictor and/or outcome domain judgement. Related but non-identical glycaemic measures were recorded separately. For longitudinal/prognostic models, baseline glycaemic predictors measured before subsequent outcome ascertainment were not automatically considered direct incorporation bias because of the temporal separation between predictor and outcome measurement.

### 2.6 Data synthesis

Clinical and methodological heterogeneity was expected across populations, prediabetes definitions, predictor sets, algorithms, and validation methods. Consequently, results were synthesised descriptively rather than meta-analysed. Formal statistical heterogeneity assessment using *I*^2^ or Cochran's *Q* was not undertaken because the included studies did not provide sufficiently comparable effect estimates for quantitative pooling. In particular, reported AUC values reflected different clinical objectives, outcome definitions, predictor structures, modelling approaches, and validation settings, rather than a common underlying effect estimate. Applying conventional heterogeneity statistics under these circumstances could therefore generate misleading quantitative precision. Model performance was summarised as reported by the primary studies, without pooling across non-comparable settings or outcome definitions. To assess whether predictor--reference-standard overlap influenced the interpretation of model discrimination, we performed a sensitivity analysis excluding current/prevalent identification studies with confirmed direct or substantial overlap between predictors and the reference standard. We then recalculated the study-level internal validation AUC range and median among studies with available AUC values. In addition, a sensitivity analysis excluding studies classified as having an overall high risk of bias according to PROBAST was conducted. The study-level AUC distribution was recalculated among the remaining studies with available AUC values to evaluate whether methodological quality influenced the overall interpretation of model discrimination.

## 3 Results

### 3.1 Study selection

The original database searches identified 4,032 records. After removal of 863 duplicates, 3,169 records underwent title and abstract screening. Seventy-five reports were sought for retrieval, of which 20 could not be retrieved; 55 full-text reports were assessed, and 19 studies met the eligibility criteria (20–38). The updated search identified 278 additional records. After removal of 110 duplicates, 168 records underwent title and abstract screening. A total of 18 reports were sought for retrieval of which eight could not be retrieved, leaving 10 full-text reports for eligibility assessment. Three reports were excluded after full-text assessment, and seven additional studies met the inclusion criteria (39–45). Overall, 26 studies were included in the review. The study selection process is shown in Figure 1.

**Figure 1:**
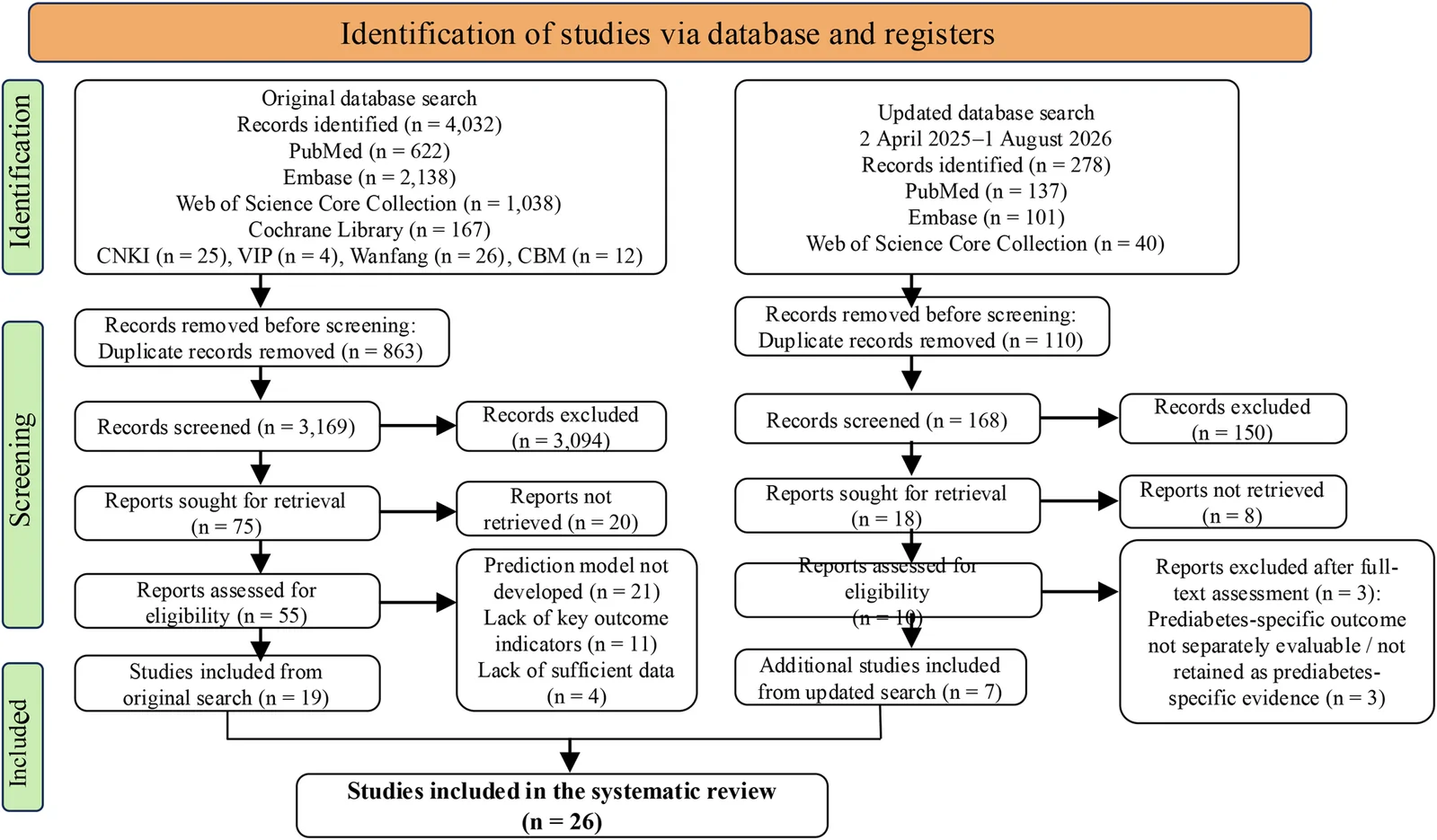
PRISMA 2020 flow diagram showing study selection from the original search and the updated search conducted during the peer review revision.

### 3.2 Study characteristics

The 26 included studies were published between 2014 and 2026 and involved a total of 435,647 participants. Study designs and data sources were heterogeneous and included retrospective and cross-sectional studies, health examination and population-based cohorts, secondary analyses of existing datasets, and multi-cohort imaging studies. Most studies enrolled adults, although some focused on specific age, sex, or clinical populations, including children, adolescents, and older men. In addition to routinely collected demographic, anthropometric, laboratory, and lifestyle data, more recent studies incorporated retinal images, inflammatory biomarkers, molecular markers, metabolomic profiles, and genomic data. Of the 26 included studies, 23 primarily addressed current/prevalent prediabetes identification. Three studies (21, 28, 32) used longitudinal/prognostic designs and were retained only as supplementary evidence. These studies evaluated future dysglycaemic outcomes, including future prediabetes, impaired fasting glucose, or related progression, rather than contemporaneous identification. They were therefore interpreted separately and were not used to draw conclusions regarding the performance, transportability, or clinical applicability of current/prevalent prediabetes identification models. However, systematic subgroup analyses evaluating model performance across demographic or socio-economic characteristics were rarely reported. The characteristics of the included studies, together with their classification as primary or supplementary evidence and their inclusion status in Figure 2, are summarised in Table 1.

**Figure 2:**
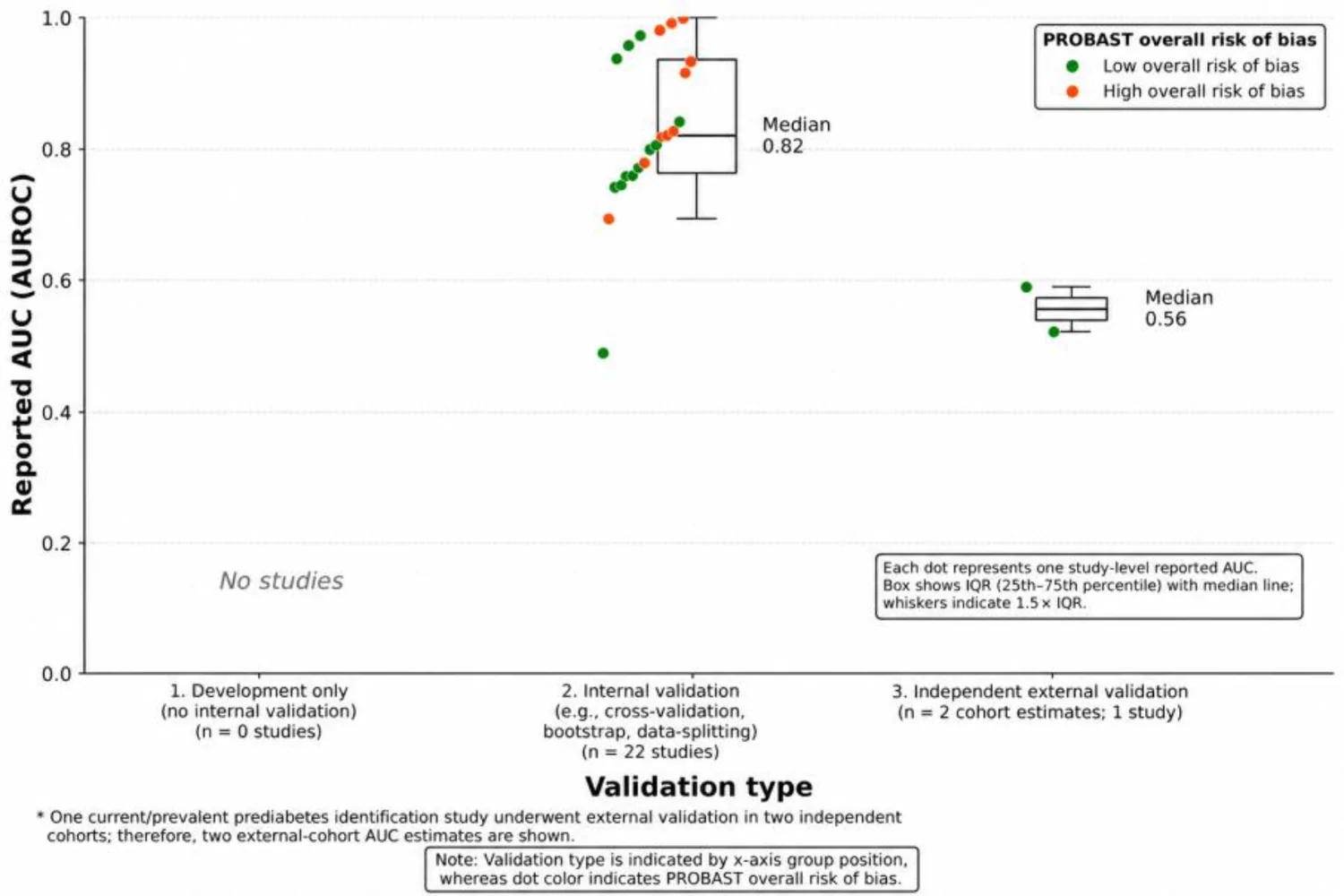
Distribution of study-level reported AUC values by validation type and overall PROBAST risk-of-bias classification. The *x*-axis represents the validation type, distinguishing development/internal validation from independent external validation, and point colours represent the overall PROBAST risk-of-bias classification of each study. Each point represents the best reported internal validation AUC from a current/prevalent prediabetes identification study where available; the two AUC estimates from the independent external cohorts of the single independently externally validated current/prevalent identification study are shown separately under independent external validation. The three longitudinal/prognostic studies (21, 28, 32) were excluded from this visualisation because they addressed temporally distinct future risk questions and were retained only as supplementary evidence.

**Table 1:** Characteristics of the included studies.

Study	Year	Country/region	Age or population	Design	Sample size (development/validation)	Validation type	Evidence role/Figure 2 status
Maalouf et al. (40)	2026	Not clearly reported	DiabHealth screening cohort	Secondary analysis	545	Internal: holdout + nested CV	Primary/Figure 2: yes
Fan et al. (43)	2026	Iraq	Non-diabetic, prediabetic, and diabetic participants	Secondary analysis	830	Internal: split + 5-fold CV	Primary/Figure 2: yes
Yesupatham et al. (39)	2025	India	Adults aged 18–60 years	Cross-sectional	199	Internal: split + 10-fold CV	Primary/Figure 2: yes
Pushpanathan et al. (42)	2025	Singapore/UK/China	Multi-cohort retinal imaging populations	Multi-cohort study	78,156	Internal + independent external (SP2, BES)	Primary/Figure 2: yes
Wang et al. (41)	2025	China	Chinese Han men aged 50–87 years	Retrospective	1,117	Internal: random split	Primary/Figure 2: yes
Matboli et al. (44)	2025	Egypt	Healthy, prediabetes, and T2DM groups	Cross-sectional	260	Internal: split + 5-fold CV	Primary/Figure 2: yes
Cha and Choi (45)	2025	South Korea	KoGES Ansan–Ansung cohort	Cross-sectional	1,819	Internal: 5-fold CV	Primary/Figure 2: yes
Li et al. (37)	2025	China	Not reported	Retrospective	8,277 (6,621/1,656)	Internal: split + 5-fold CV	Primary/Figure 2: yes
Yang et al. (38)	2025	China	43.85 ± 13.34	Retrospective	6,153 (4,287/1,866)	Internal: random split	Primary/Figure 2: yes
Zhang et al. (36)	2024	China	Not reported	Retrospective	59,259 (32,810/26,449)	Internal: split + 10-fold CV	Primary/Figure 2: yes
Nguyen et al. (35)	2024	South Korea	Men aged ≥30 years	Retrospective	1,527 (1,069/458)	Internal: random split	Primary/Figure 2: yes
Heo (33)	2024	South Korea	27.20 ± 4.78	Retrospective	823 (577/246)	Internal: split + 5-fold CV	Primary/Figure 2: yes
Lu et al.(34)	2024	Canada	63.0 (range 18.0–90.0)	Retrospective	6,414 (4,810/1,604)	Internal: random split	Primary/Figure 2: yes
Cui et al. (32)	2024	China	Development: IFG 56.0; non-IFG 55.0; validation: IFG 63.0; non-IFG 62.5	Retrospective	1,855 (1,438/417)	Internal: split + 5-fold CV	Supplementary/Figure 2: no
Chen et al. (31)	2024	China	Not reported	Retrospective	5,127	Internal: split + 5-fold CV	Primary/Figure 2: yes
Vîrgolici and Virgolici (30)	2023	Romania	Not reported	Retrospective	125 (100/25)	Internal: random split	Primary/Figure 2: no (AUC NR)
Ou et al. (29)	2023	China	Non-diabetes 41.74; prediabetes 51.28; diabetes 56.79	Retrospective	1,428	Internal: split + 10-fold CV	Primary/Figure 2: yes
Hu et al. (28)	2023	China	41.02 ± 12.10	Retrospective	184,188 (92,177/92,011)	Internal: development/validation split	Supplementary/Figure 2: no
Gong et al. (27)	2023	China	Not reported	Cross-sectional	5,310 (4,248/1,062)	Internal: random split	Primary/Figure 2: yes
Kushwaha et al. (26)	2022	India	5–19 years	Retrospective	42,688 (30,735/11,953)	Internal: split + 10-fold CV	Primary/Figure 2: yes
Dong et al. (25)	2022	China	18–84 years	Cross-sectional	1,857 (1,238/619)	Internal: split + 5-fold CV	Primary/Figure 2: yes
Li et al. (24)	2021	China	Not reported	Cross-sectional	1,710 (1,026/684)	Internal: split + 10-fold CV	Primary/Figure 2: yes
Abbas et al. (23)	2021	Qatar	18–86 years	Cross-sectional	7,268 (5,814/1,454)	Internal: split + 10-fold CV	Primary/Figure 2: yes
Wang et al. (22)	2020	China	Not reported	Retrospective	2,251/663	Internal: 5-fold CV	Primary/Figure 2: yes
Wang et al. (21)	2020	Japan	Development 43.52 ± 8.50; validation 43.40 ± 8.45	Retrospective	11,113 (8,296/2,817)	Internal: development/validation split	Supplementary/Figure 2: no
Choi et al. (20)	2014	South Korea	Normal 41.9 ± 0.5; prediabetes 52.5 ± 0.6	Retrospective	3,134/1,551	Internal: split + 10-fold CV	Primary/Figure 2: yes

CV, cross-validation; IFG, impaired fasting glucose. Ages are reported as mean ± standard deviation or as stated in the source article. A slash without parentheses indicates development and validation samples as reported by the original study. Primary = current/prevalent identification; Supplementary = longitudinal/prognostic evidence. Figure 2 includes primary studies with a reported AUC; Vîrgolici et al. (30) was not plotted because AUC was not reported, and supplementary studies (21, 28, 32) were excluded.

### 3.3 Model development and performance

Across all included evidence, the 26 studies reported 110 models; this total includes the three longitudinal/prognostic studies retained as supplementary evidence. Descriptive counts of algorithms and predictors in the following summarise all included evidence unless otherwise specified, whereas conclusions regarding clinical performance, transportability, and implementation readiness are restricted to the current/prevalent identification evidence. These models spanned a broad range of machine learning and statistical learning approaches. Logistic regression was evaluated in 17 studies, random forest in 14, XGBoost in 13, support vector machines in 12, and neural network or deep learning approaches in 11. Other approaches included decision trees, gradient boosting machines, LightGBM, CatBoost, AdaBoost, Extra Trees, *k*-nearest neighbours, naive Bayes, linear and quadratic discriminant analysis, Cox-based models, and newer architectures such as TabPFN, HyperTab, vision transformers, and interpretable deep learning models.

Common predictors included demographic characteristics, anthropometric measurements, glycaemic biomarkers, lipid-related variables, family history, and lifestyle factors. More recent studies also incorporated specialised inputs, including inflammatory biomarkers, retinal fundus images, molecular markers, metabolomic profiles, and genomic variants. Study-by-study assessment identified substantial heterogeneity in the relationship between glycaemic predictors and outcome definitions (Supplementary Table S4). In some current/prevalent classification studies, a glycaemic measurement contributing directly to the reference standard was also included among the model predictors, creating a clear risk of incorporation bias. Other studies used related but non-identical glycaemic measurements. In the longitudinal/prognostic studies, baseline predictors were temporally separated from subsequent outcome ascertainment and were therefore considered separately when evaluating potential predictor–outcome incorporation.

Among the 26 included studies, three studies (11.5%) showed confirmed direct or substantial predictor–reference-standard overlap, four studies (15.4%) showed ambiguous or potential overlap, and 19 studies (73.1%) showed no identified overlap based on the available information. Direct or substantial overlap primarily involved glycaemic measurements such as fasting glucose or HbA1c being used both as model predictors and as components of the reference standard or class label definition.

The magnitude of possible AUC inflation could not be quantified uniformly because most primary studies did not report otherwise identical models with and without the overlapping glycaemic predictors. Where within-study comparisons were available, the observed differences were reported descriptively. In Maalouf et al. (40), the mean AUC was 0.734 for the all-features panel including HbA1c and 0.734 for the all-biomarkers panel excluding HbA1c, while the final non-glycaemic inflammatory model achieved a mean AUC of 0.743. In Matboli et al. (44), the molecular-only model achieved an AUC of 0.9956 compared with 0.9989 for the combined molecular/biochemical model, an absolute difference of 0.0033. However, because these feature sets differed in multiple variables, these contrasts should not be interpreted as causal estimates of AUC inflation attributable specifically to glycaemic predictor–outcome overlap.

As a sensitivity analysis, we excluded studies with confirmed direct or substantial predictor–reference-standard overlap (30, 39, 44). Vîrgolici et al. (30) did not contribute an AUC estimate to the study-level visualisation because an AUC was not reported. After additionally excluding Yesupatham et al. (39) and Matboli et al. (44), 20 current/prevalent identification studies with available internal validation AUC values remained. The study-level AUC range decreased from 0.49–0.9989 to 0.49–0.9913, and the median AUC decreased modestly from 0.821 to 0.813. Thus, exclusion of studies with confirmed direct or substantial overlap reduced the upper end of the reported discrimination range but did not materially change the overall interpretation that high internal validation performance coexists with limited external validation and substantial methodological heterogeneity. Details of this sensitivity analysis are provided in Supplementary Table S6.

Reported AUC values from development or internal validation procedures ranged from 0.49 to 0.9989 across heterogeneous modelling tasks and validation settings. The lowest reported validation AUC (0.49) was observed for the random forest model in the study by Heo et al. (33). At the upper end of the range, Matboli et al. (44) reported an AUC of 0.9989 for a combined Extra Trees model; however, this estimate represented overall multiclass discrimination across healthy, prediabetes, and diabetes-related categories rather than a prediabetes-specific AUC. Therefore, direct comparison of AUC values across studies should be interpreted cautiously because populations, outcome structures, predictor sets, and validation designs differed substantially. When model performance was examined according to validation type, the highest AUC values were predominantly reported in development or internal validation settings. Among the 110 reported models, 109 were evaluated only using development/internal validation strategies, whereas one additionally underwent independent external validation. The externally validated model demonstrated lower discrimination performance, with AUC values of 0.590 and 0.523 in two independent external cohorts, compared with the corresponding internal evaluation. For descriptive visualisation of the primary current/prevalent identification evidence, one best-reported internal validation AUC per study was plotted where available (22 studies). The three longitudinal/prognostic studies (21, 28, 32) were excluded from this visualisation of current/prevalent identification evidence and retained only as supplementary evidence. The two external cohort AUC estimates from the single current/prevalent identification study that underwent independent external validation are shown separately in Figure 2. In Figure 2, the *x*-axis represents validation type, whereas point colours represent the overall PROBAST risk-of-bias classification. This allows reported discrimination to be interpreted jointly according to validation type and methodological quality.

Independent external validation was reported in one study (42). In the study by Pushpanathan et al., the prediabetes-versus-healthy model achieved an internal AUROC of 0.746 (95% CI 0.733–0.759), which decreased to 0.590 (95% CI 0.569–0.611) in the SP2 external cohort and 0.523 (95% CI 0.488–0.557) in the BES external cohort. This decline highlights the importance of distinguishing development or internal validation performance from transportability to independent populations.

A complementary study-level visualisation is provided in Supplementary Figure S1, which displays reported AUC estimates with 95% confidence intervals where available, stratified by validation type and colour-coded by overall PROBAST risk-of-bias classification. This figure further illustrates the heterogeneity of reported discrimination performance across studies and highlights the attenuation observed in the only study that underwent independent external validation. For studies in which confidence intervals were not reported, only the point estimate is shown.

XGBoost was frequently evaluated and showed strong discrimination in several individual datasets (24–26, 37, 38). However, its performance varied across studies, and the available evidence does not establish universal superiority over other algorithms. Several studies also compared ML approaches with logistic regression or other conventional methods, but the magnitude of improvement varied across datasets and evaluation strategies. Given the limited independent external validation and sparse assessment of calibration and clinical utility, whether more complex ML models provide meaningful incremental benefit over simpler conventional approaches remains uncertain.

Reporting and implementation of model development procedures remained heterogeneous. Some newer studies explicitly embedded pre-processing, feature selection, class imbalance handling, and hyperparameter optimisation within training folds or nested cross-validation, thereby reducing the risk of information leakage (40, 43, 44). In contrast, other studies either provided insufficient detail or performed parts of predictor selection before cross-validation, which may have allowed information from validation data to influence model development (45). Accordingly, the potential for optimistic performance estimates due to information leakage could not be excluded across the evidence base.

### 3.4 Predictors and data requirements

Models used between one and 129 reported predictors or input features. Frequently reported predictors included age, sex, education, family history of diabetes, fasting plasma glucose, glycated haemoglobin, body mass index, triglycerides, waist circumference, diet, physical activity, and alcohol consumption. Age, body mass index, waist circumference, glycaemic measures, and lipid-related variables remained among the commonly reported predictors. These variables are generally compatible with routine primary care or health examination data.

Some studies incorporated specialised inputs, including electrocardiograms, tongue images, skinfold measurements, detailed dietary variables, inflammatory biomarkers, retinal fundus photographs, molecular markers, metabolomic profiles, and genomic variants (22, 24, 26, 33, 40, 42, 44, 45). Although such inputs may provide additional biological or phenotypic information, they may require specialised imaging equipment, laboratory assays, data processing infrastructure, or technical expertise, which could limit immediate feasibility in routine primary care settings. Detailed predictors, predictor selection methods, and missing data handling are reported in Supplementary Material 2.

### 3.5 Validation and reporting practices

Most included studies relied on internal validation, commonly using random train–test splits, cross-validation, or combinations of these approaches. Only one of the 26 studies reported independent external validation (1/26, 3.8%), using two independent external cohorts (42). In that study, discrimination for prediabetes was lower in both external cohorts than in internal testing, highlighting the limited transportability that may be apparent only when models are evaluated in independent populations. The remaining studies relied exclusively on development or internal validation data.

Calibration was reported in a minority of studies and was assessed using heterogeneous approaches, including calibration plots, calibration slopes/intercepts, Hosmer–Lemeshow tests, or comparisons between predicted and observed risks. However, most studies focused primarily on discrimination metrics such as AUC, while comprehensive assessment of calibration and clinical utility was uncommon. Explicit assessment of clinical utility using decision curve analysis was reported in only three of the 26 included studies (11.5%). Among the primary current/prevalent identification studies, Zhang et al. (36) evaluated seven machine learning models using decision curve analysis and reported clinical benefit for the CatBoost model across the evaluated threshold probability range. Two longitudinal/prognostic studies retained as supplementary evidence also reported decision curve analysis. Hu et al. (28) evaluated net benefit in both the training and validation cohorts across alternative threshold probabilities, whereas Cui et al. (32) used decision curve analysis to assess the clinical utility of the XGBoost model and reported potential net benefit when higher-risk individuals identified by the model were targeted for intervention. No explicit decision curve or net benefit assessment was identified in the remaining included studies. These findings indicate that evidence of clinical utility remains sparse and is insufficient to establish whether the reported gains in discrimination translate into improved clinical decision-making. Missing data handling also varied across studies: approaches included imputation, complete-case or listwise deletion, exclusion of participants or images with incomplete data, and, in some studies, incompletely described procedures. The timing of missing data handling relative to model development and validation was also not consistently reported. Study-level missing data methods and their reported relationship to the validation process are summarised in Supplementary Table S7. Reporting of pre-processing, feature selection, class imbalance handling, and hyperparameter optimisation was similarly heterogeneous. Full details of model development, validation, performance, and missing data handling are provided in Supplementary Material 2.

### 3.6 Risk of bias and applicability

Based on the PROBAST assessment, 14 of the 26 included studies were judged to have an overall low risk of bias, whereas 12 studies (46.2%) were judged to have an overall high risk of bias. At the domain level, high-risk judgements were most frequent in the analysis domain (12/26, 46.2%), followed by the outcome domain (11/26, 42.3%), predictor domain (9/26, 34.6%), and participant domain (7/26, 26.9%). Detailed domain-level results are summarised in Table 2, and study-level risk-of-bias and applicability assessments are provided in Supplementary Table S3. A sensitivity analysis was performed excluding the 12 studies judged to have an overall high risk of bias according to PROBAST. Among studies with available AUC estimates, the reported AUC range changed from 0.490 to 0.9989 in the primary analysis to 0.490–0.9731 after exclusion of high-risk studies, while the median AUC decreased from 0.820 to 0.800. These findings indicate that exclusion of studies with a high risk of bias reduced the upper range of reported discrimination but did not materially change the overall interpretation that current machine learning models demonstrate promising discrimination but insufficient evidence for routine primary care implementation.

**Table 2:** Distribution of PROBAST domain-level risk of bias among included studies.

PROBAST domain	Studies with high risk, *n* (%)	Main concerns
Participants	7/26 (26.9)	Selected or restricted study populations and limited representativeness
Predictors	9/26 (34.6)	Unclear predictor selection or assessment procedures and predictor–outcome overlap in some studies
Outcome	11/26 (42.3)	Heterogeneous outcome definitions or ascertainment and direct predictor–reference-standard overlap causing potential incorporation bias
Analysis	12/26 (46.2)	Incomplete model development reporting, small effective samples, limited validation, non-nested feature selection or pre-processing, and potential optimism

Methodological concerns in the analysis domain were related to incomplete reporting or implementation of model development procedures, small effective sample sizes in some studies, limited independent validation, and the potential for optimistic performance estimates arising from feature selection or other pre-processing conducted outside the validation framework.

The supplementary ML-specific audit showed substantial variation in the handling and reporting of modelling workflows. Some studies explicitly restricted pre-processing, feature selection, class imbalance handling, or hyperparameter optimisation to training data or nested resampling procedures, whereas others either performed feature selection or pre-processing before cross-validation or did not report sufficient information to exclude leakage. Clear evidence of information leakage, pre-processing or feature selection conducted outside the validation framework, or hyperparameter tuning/model selection involving evaluation data was treated as a high-risk methodological concern and contributed to the PROBAST analysis domain judgement, whereas insufficiently reported procedures were recorded as unclear. Supplementary Table S5 explicitly identifies the study-level leakage/optimism concerns and shows how these findings informed the corresponding analysis domain judgement.

Outcome domain concerns included heterogeneity in the definition and ascertainment of prediabetes and study-specific incorporation bias. In current/prevalent identification studies where the same glycaemic measurement contributed to both model prediction and the reference standard, this overlap was considered a high-risk concern and was incorporated into the corresponding PROBAST judgement. Detailed study-level assessments of predictor–reference-standard overlap are provided in Supplementary Table S4. Predictor domain concerns were associated with unclear predictor selection or assessment procedures and predictor–outcome overlap in some studies. Participant domain concerns mainly reflected selected or restricted study populations and limited representativeness. With regard to applicability, eight studies (8/26, 30.8%) were judged to have overall high applicability concerns, most commonly because of limitations in participant representativeness or differences between the study population and the intended screening context.

## 4 Discussion

### 4.1 Principal findings

This systematic review identified a heterogeneous and rapidly developing evidence base for machine learning-supported identification of prediabetes. Following the updated search through 1 August 2026, 26 studies involving 435,647 participants and 110 reported models were included. Many models demonstrated promising discrimination performance, and several relied on variables that are routinely available in primary care. However, these development or internal validation results should not be interpreted as evidence of implementation readiness. High discrimination estimates obtained from development datasets primarily reflect performance under specific study conditions and may not translate directly to routine primary care practice without independent validation and evaluation of clinical utility. Twelve of the 26 studies (46.2%) were judged to have an overall high risk of bias, and independent external validation remained uncommon.

The core evidence in this review comprised 23 studies evaluating current/prevalent prediabetes identification. Three longitudinal/prognostic studies were retained only as supplementary evidence and interpreted separately because they addressed future dysglycaemic outcomes rather than contemporaneous identification. Accordingly, these studies were not used to support conclusions regarding the discrimination, transportability, or implementation readiness of current/prevalent identification models. Future studies should further investigate longitudinal machine learning approaches incorporating repeated measurements, temporal changes, and follow-up data to improve prediction of future prediabetes and related dysglycaemic progression.

Only one study reported independent external validation (1/26, 3.8%). In that study, the AUROC for distinguishing prediabetes from healthy status decreased from 0.746 (95% CI 0.733–0.759) in internal evaluation to 0.590 (95% CI 0.569–0.611) and 0.523 (95% CI 0.488–0.557) in two independent external cohorts. This reduction illustrates the distinction between performance observed during model development and transportability to different populations and settings. Models evaluated only within their source datasets may capture population-specific patterns and may therefore perform poorly when applied in other primary care populations or healthcare systems.

Reported development or internal validation AUC values ranged from 0.49 to 0.9989, but this wide range should be interpreted cautiously because the included studies differed substantially in population characteristics, outcome definitions, predictor sets, class balance, modelling tasks, and validation strategies. The lowest reported validation AUC (0.49) was observed for a random forest model in the study by Heo et al. (33), whereas the highest value (0.9989) was reported by Matboli et al. (44) for an overall multiclass model rather than a prediabetes-specific binary outcome. These values are therefore not directly comparable indicators of model quality.

Overall, the updated evidence suggests that discrimination alone is insufficient for judging the clinical value of prediabetes models. A modest gain in AUC within a development dataset may be less important than reliable calibration, acceptable sensitivity and specificity at clinically relevant thresholds, reproducible performance in independent populations, and evidence of net clinical benefit. The analysis by validation type further highlighted that high discrimination values should be interpreted in the context of validation robustness rather than considered standalone indicators of clinical utility. The addition of PROBAST-based colour coding to Figure 2 further emphasises that reported discrimination should not be interpreted independently of methodological quality, as apparently strong AUC values may still arise from studies at high risk of bias. The current evidence base therefore supports continued model development and external validation rather than routine implementation in primary care.

Another important consideration is algorithmic fairness and subgroup generalisability. Although demographic, clinical, and lifestyle characteristics were frequently used as predictors, most included studies reported only overall model performance and did not evaluate whether model accuracy, discrimination, or calibration differed across age, sex, ethnicity, socio-economic groups, or geographic populations. Therefore, the extent to which current/prevalent prediabetes identification models perform equitably across diverse populations remains uncertain. Future studies should incorporate pre-specified subgroup analyses, fairness metrics, and external validation across diverse populations to ensure equitable implementation in primary care.

### 4.2 Implications for family medicine and primary care

For primary care, the most useful models may not necessarily be the most complex. Age, body mass index, waist circumference, blood pressure, fasting glucose, glycated haemoglobin, lipids, family history, and lifestyle factors can be collected during routine care and incorporated into electronic health records. Models based primarily on these routinely available variables may be easier to integrate into existing screening pathways and could support targeted biochemical testing, structured lifestyle counselling, and risk-based follow-up. Non-laboratory approaches may also be useful where biochemical testing is less accessible (23, 25, 26).

However, several more recent studies incorporated specialised inputs, including inflammatory or molecular biomarkers, retinal fundus images, metabolomic profiles, and genomic variants (40, 42, 44, 45). Although these data sources may provide additional biological or phenotypic information, they may also require specialised assays, imaging equipment, computational infrastructure, or technical expertise. Their incremental predictive value should therefore be weighed against feasibility, cost, accessibility, and workflow burden before they are considered for routine primary care screening.

Clinical usefulness also depends on the intended care pathway. A model should specify who is assessed, when the score is calculated, which threshold triggers action, and what clinical action follows. Potential actions include confirmatory biochemical testing, referral, structured lifestyle intervention, or intensified follow-up. Threshold selection should explicitly consider the trade-off between sensitivity and specificity: False-positive classifications may increase workload, patient burden, and unnecessary testing, whereas false-negative classifications may delay preventive intervention. These consequences should be evaluated alongside discrimination, calibration, and, where possible, decision curve or net benefit analyses.

A model developed in a hospital database, health examination cohort, or other source population should not be assumed to perform similarly in community or family practice populations. Differences in patient characteristics, disease prevalence, laboratory methods, healthcare access, and clinical workflows can alter both calibration and discrimination. Independent external validation was reported in only one of the 26 included studies (3.8%), and that study showed lower discrimination in two external cohorts than in internal evaluation. The transportability of most current machine learning models therefore remains uncertain. External validation and, where necessary, recalibration in representative primary care populations are essential before routine clinical implementation.

Health equity is also an important consideration when assessing model generalisability. Models developed from datasets with limited representation of particular socio-economic, ethnic, age, sex, or geographic groups may perform differently across populations because of differences in disease prevalence, healthcare access, health behaviours, data completeness, and predictor distributions. Future validation studies should therefore report performance across clinically relevant subgroups and examine whether discrimination, calibration, sensitivity, specificity, and clinical utility remain consistent across diverse populations. Where systematic performance differences are identified, model recalibration or redesign may be required before implementation.

The distinction between current/prevalent prediabetes identification and longitudinal/prognostic prediction is also important for clinical interpretation. Models designed to identify current/prevalent prediabetes may support targeted screening or confirmatory testing, whereas longitudinal/prognostic models are intended to estimate future dysglycaemic outcomes over a defined follow-up period. These two objectives require different study designs, temporal structures, outcome definitions, and validation strategies and should not be interpreted interchangeably. Future longitudinal studies should therefore report prediction horizons clearly and validate longitudinal/prognostic models using appropriately defined follow-up periods.

### 4.3 Methodological reliability and implementation

Discrimination alone is insufficient to determine whether a model improves care. Future studies should report calibration, clinically relevant thresholds, sensitivity and specificity at those thresholds, and decision curve analysis. In primary care screening scenarios, the optimal balance between sensitivity and specificity should be determined according to the intended clinical objective, disease prevalence, and available healthcare resources. Decision curve analysis and net benefit evaluation may help determine whether a model offers clinically meaningful improvement over alternative screening strategies. Importantly, future studies should demonstrate incremental value beyond established approaches, including logistic regression, validated clinical risk scores, and parsimonious screening models. Such comparisons should consider calibration, decision curve analysis, net benefit, and implementation burden rather than relying solely on small improvements in discrimination metrics.

#### 4.3.1 Call to action: clinical utility and fairness evaluation

For future primary care studies, evidence of clinical utility should extend beyond discrimination alone and include three complementary components. First, sensitivity and specificity should be reported at pre-specified clinically relevant decision thresholds. Second, decision curve analysis should demonstrate positive net benefit across those thresholds compared with existing screening strategies and treat-all/treat-none alternatives. Third, prospective implementation or impact evaluation should determine whether model-guided use improves or meaningfully supports clinical decision-making in real-world primary care workflows without unacceptable burden or unintended harm. Together, these components provide a more clinically informative assessment than AUC alone.

Fairness assessment should likewise extend beyond reporting overall model performance. Future studies should evaluate performance across clinically relevant demographic and clinical subgroups and consider complementary fairness metrics, including demographic parity, equalised odds, and subgroup calibration. Demographic parity assesses whether positive classifications or resulting actions occur at comparable rates across groups; equalised odds evaluate whether true-positive and false-positive rates are comparable across groups; and subgroup calibration assesses agreement between predicted and observed risk within each subgroup. Because no single fairness metric is universally sufficient, these measures should be interpreted in relation to baseline disease prevalence, the intended clinical use, and the potential consequences of model-guided decisions.

To provide a more operational definition of clinical readiness, we propose several pragmatic criteria that should be considered before a machine learning model is adopted for routine primary care use. These criteria encompass external validation and transportability, calibration, clinical utility, prospective implementation, health economic value, subgroup performance, and methodological robustness, as summarised in Table 3.

**Table 3:** Proposed pragmatic criteria for clinical readiness of machine learning models in primary care.

Domain	Proposed pragmatic readiness criterion	Suggested assessment or benchmark
External validation and transportability	Performance should be reproduced in multiple independent populations representative of the intended primary care setting	Validation in at least three independent populations may provide stronger evidence of transportability; this is a pragmatic benchmark rather than a universal requirement
Calibration	Calibration should be demonstrated in the intended target population	Calibration plot with a smoothed calibration curve, calibration slope (ideal value 1), and calibration-in-the-large (ideal value 0), with estimates and confidence intervals interpreted in the intended target population and clinical context rather than against a universal numerical cut-off
Clinical utility	The model should provide clinically meaningful benefit beyond existing screening approaches	Threshold-specific sensitivity and specificity at pre-specified clinically relevant decision thresholds, together with decision curve analysis demonstrating positive net benefit compared with existing screening strategies and treat-all/treat-none alternatives
Prospective implementation	The model should function effectively within real-world primary care workflows	Prospective implementation or impact evaluation demonstrating support for clinical decision-making without unacceptable workflow burden or unintended harm
Health economic value	Implementation should be economically acceptable relative to current practice	Assessment of implementation costs, resource use, and cost-effectiveness compared with existing screening strategies
Equity, subgroup performance, and fairness assessment	Performance should remain acceptable across clinically relevant population subgroups	Evaluation of performance across relevant demographic and clinical subgroups, with reporting of fairness indicators such as demographic parity, equalised odds, and subgroup calibration
Methodological robustness	Model development and validation should minimise avoidable optimism and information leakage	Transparent, leakage-resistant pre-processing, feature selection, tuning, and validation procedures, with appropriate separation of development and evaluation data

These criteria are proposed as pragmatic readiness benchmarks rather than universally mandated regulatory thresholds. Their interpretation should consider the intended population, clinical pathway, healthcare resources, and model use.

#### 4.3.2 Practical checklist for local implementation

For primary care clinicians and local decision-makers considering implementation of a machine learning model, the criteria in Table 3 can be translated into a brief practical checklist: (1) Has the model been externally validated in a population sufficiently similar to the intended local population? (2) Has calibration been evaluated and found acceptable in the target setting? (3) Are sensitivity, specificity, and net benefit reported at pre-specified clinically relevant decision thresholds? (4) Has the model undergone prospective implementation or impact evaluation within a real-world primary care workflow? (5) Are the required predictors, data systems, equipment, personnel, and implementation costs feasible within the local setting? (6) Is model performance acceptable across relevant demographic and clinical subgroups, with appropriate assessment of fairness? (7) Are model development, validation, updating, and data processing procedures sufficiently transparent and methodologically robust? A high AUC alone should not be considered sufficient evidence of implementation readiness when important checklist items remain unaddressed. If key criteria are not met, further local validation, recalibration, or prospective evaluation should be considered before routine deployment.

Reliable assessment of clinical utility also depends on rigorous model development procedures. A single random training–testing split may produce optimistic estimates, particularly when pre-processing, feature selection, class imbalance handling, or hyperparameter tuning is performed before rather than within the validation procedure. Some newer studies reduced this risk by restricting predictor selection or pre-processing to training folds or by using nested cross-validation (40, 43). In contrast, feature selection conducted on the full dataset before cross-validation may allow information from validation observations to influence model development, thereby inflating apparent performance (45). Transparent reporting of predictor selection, missing data handling, predictor timing, class balancing, feature engineering, hyperparameter optimisation, and the exact sequence of validation procedures is therefore essential. Sample size is another important consideration when interpreting ML model performance. Six included studies had fewer than 1,000 participants (30, 33, 39, 40, 43, 44), including three with fewer than 300 participants (30, 39, 44). Small sample size does not necessarily render an ML model invalid, because adequacy depends on the number of observations within each outcome class, predictor dimensionality, model complexity, class imbalance, tuning procedure, and validation strategy. However, complex or high-dimensional models developed in small datasets are more vulnerable to overfitting, unstable feature or model selection, and optimistic performance estimates, particularly when the available sample is further divided into training, validation, and test subsets. Very high internal validation AUCs reported in some small-sample studies should therefore be interpreted cautiously and in conjunction with other methodological concerns, including information leakage and predictor–reference-standard overlap, rather than attributed to sample size alone. Strong internal performance from a small dataset should be regarded as preliminary until reproducibility is demonstrated in adequately sized independent populations.

The updated PROBAST assessment showed that methodological concerns were most frequent in the analysis domain, followed by the outcome and predictor domains. These findings indicate that limitations were often related to how models were developed, validated, and evaluated rather than to algorithm choice alone. Although PROBAST provides a structured framework for assessing prediction model studies, conventional PROBAST does not explicitly capture all risks arising from complex machine learning workflows. We therefore supplemented the PROBAST assessment with a structured ML-specific audit informed by PROBAST + AI principles (19). This audit examined data leakage, the placement of pre-processing and feature selection procedures relative to resampling, class imbalance handling, separation of hyperparameter optimisation from final evaluation, and the use of nested cross-validation or equivalent procedures. These findings were incorporated into the PROBAST analysis domain judgements rather than treated as a separate performance metric. The audit reinforced that apparently strong discrimination may be optimistic when feature selection, pre-processing, or model tuning is influenced by information from evaluation data. Conversely, workflows that restrict these procedures to training folds and use nested or otherwise appropriately separated validation provide stronger protection against optimism.

Another important methodological concern is incorporation bias in current/prevalent prediabetes identification models. The study-by-study assessment showed that the degree of predictor–outcome overlap varied substantially. In some studies, glycaemic variables contributing directly to the reference standard were also used as model inputs, whereas other studies used related but non-identical glycaemic markers. Such direct overlap can allow the model to partially reproduce the diagnostic rule rather than identify additional independent predictive information, potentially producing optimistic discrimination. However, the magnitude of this effect could not be quantified consistently because most studies did not report otherwise identical models with and without the overlapping predictors. Available within-study comparisons did not show a uniform effect: for example, Maalouf et al. (40) reported the same mean AUC (0.734) for an all-features panel including HbA1c and an all-biomarkers panel excluding HbA1c, whereas Matboli et al. (44) reported AUCs of 0.9956 for the molecular-only model and 0.9989 for the combined molecular/biochemical model. These differences cannot be attributed specifically to incorporation bias because the compared models differed in multiple features. Consequently, very high AUC values from models with direct predictor–reference-standard overlap should be interpreted cautiously. Our sensitivity analysis further showed that excluding studies with confirmed direct or substantial predictor–reference-standard overlap modestly reduced the upper range and median of study-level internal validation AUCs but did not materially alter the main conclusions of the review. This suggests that incorporation bias may contribute to some of the most optimistic discrimination estimates, while the broader concerns regarding limited external validation, calibration, and clinical utility remain even after these studies are excluded. Future studies should report sensitivity analyses excluding variables that directly contribute to the diagnostic definition.

Interpretation of reported model performance should also consider validation design and algorithmic context. Because independent external validation was available in only one study, most reported performance estimates still require confirmation in independent populations and healthcare settings. The decline in discrimination observed in the externally validated study illustrates how performance may attenuate when a model is transferred beyond its development population. Although XGBoost demonstrated strong performance in several included studies, current evidence does not support considering it universally superior to other algorithms. Algorithm performance depended on population characteristics, predictor availability, data quality, sample size, modelling objective, and validation strategy. Therefore, model selection should be based on reproducibility, calibration, clinical utility, interpretability, and implementation feasibility rather than discrimination alone.

### 4.4 Contribution in relation to previous reviews

The earlier systematic review of machine learning approaches for prediabetes screening included five studies and highlighted heterogeneity and validation-related limitations affecting generalisability (15). The present review substantially expands this evidence base by including 26 studies, 435,647 participants, and 110 reported models, allowing a broader evaluation of contemporary machine learning approaches and their potential relevance to primary care screening.

Beyond increasing the number of included studies, this review extends previous work by systematically evaluating methodological quality using PROBAST and by examining issues that directly influence clinical translation, including external validation, calibration, clinical utility assessment, predictor availability, and model-development transparency. Our findings indicate that although many models achieved promising discrimination within development or internal validation settings, evidence supporting transportability and routine implementation remains limited.

This review also provides a more balanced assessment of algorithmic performance. Although algorithm-specific performance summaries may appear useful, quantitative comparison of median AUC values across algorithm categories was considered inappropriate because algorithms were evaluated under substantially heterogeneous prediction objectives, populations, predictor sets, outcome definitions, and validation strategies. Therefore, observed differences in discrimination may reflect study design and validation context rather than algorithm choice alone. Consistent with this consideration, our findings do not support the assumption that more complex algorithms are inherently superior. XGBoost and other advanced machine learning methods demonstrated strong performance in some individual datasets; however, their performance varied substantially across studies, and current evidence does not establish universal superiority over conventional approaches such as logistic regression or simpler risk models.

By integrating methodological assessment with considerations of primary care feasibility, this review highlights that successful implementation of machine learning models requires not only adequate discrimination but also reliable validation, interpretability, clinical usefulness, and compatibility with existing healthcare workflows.

### 4.5 Strengths and limitations

Strengths of this review include prospective registration, a comprehensive original search across eight English- and Chinese-language databases, an updated search during peer review revision, independent screening and data extraction, CHARMS-informed data collection, and structured risk-of-bias assessment using PROBAST. The focus on primary care implementation allowed model discrimination to be considered alongside calibration, external validity, predictor availability, methodological transparency, health equity, and workflow requirements.

Several limitations should be considered. First, substantial clinical and methodological heterogeneity precluded meaningful quantitative pooling. Included studies differed in population characteristics, definitions of prediabetes, predictor sets, modelling objectives, validation strategies, and reported performance metrics. Consequently, reported AUC values should not be interpreted as directly comparable estimates of model quality. Because conventional heterogeneity statistics such as *I*^2^ and Cochran's *Q* require comparable effect estimates derived from a sufficiently homogeneous clinical and methodological framework, we considered these measures not applicable to the present synthesis. Applying such statistics across prediction studies with different clinical objectives, outcome definitions, predictor structures, and validation settings could create a misleading impression of quantitative comparability. Second, reporting of model development procedures was incomplete or inconsistent in several primary studies. Information on missing data handling, pre-processing, feature selection, class imbalance management, hyperparameter optimisation, calibration, and the ordering of these procedures relative to validation was not always available. Because this review relied on published reports and supplementary materials, unreported analytical decisions and potential information leakage could not be fully evaluated. Sample size adequacy was also difficult to assess consistently because several primary studies did not provide sufficient information on class-specific sample sizes, effective event counts, or the relationship between sample size, predictor dimensionality, and model complexity. Consequently, although small effective samples were considered within the PROBAST analysis domain assessment, we could not apply a uniform quantitative sample size adequacy criterion across all included ML studies. Third, although conventional PROBAST was supplemented with a structured ML-specific methodological audit informed by PROBAST + AI principles (19), this additional assessment depended on the methodological details reported in the primary studies. Incomplete reporting of pre-processing, feature selection, class imbalance handling, hyperparameter optimisation, and their temporal relationship to validation therefore limited our ability to exclude all ML-specific sources of bias. Fourth, some studies used fasting glucose, glycated haemoglobin, or related glycaemic measures as predictors, while the same or closely related variables contributed to the definition of prediabetes. This predictor–outcome overlap creates the possibility of incorporation bias and may inflate apparent discrimination. In particular, three studies showed confirmed direct or substantial overlap. In Vîrgolici et al. (30), serum glucose and other ATP III components were used both to construct the output label and as model predictors. In Yesupatham et al. (39), HbA1c was used both to define control/prediabetes status and as a model input. In Matboli et al. (44), fasting glucose, postprandial glucose, and HbA1c were included as predictors, while glycaemic/clinical status contributed to class assignment. These examples illustrate how a model may partially access information embedded within the reference standard, potentially contributing to optimistic discrimination. Although we examined this issue on a study-by-study basis, the magnitude of AUC inflation attributable specifically to predictor–reference-standard overlap could not be estimated for most studies because paired models with and without the overlapping glycaemic variables were generally not reported. Because predictor timing and outcome ascertainment were not always reported in sufficient detail, the magnitude of this bias could not be quantified. Another potential limitation is publication bias. Studies reporting better-performing machine learning models may be more likely to be published than studies showing limited discrimination or unsuccessful model development. However, formal statistical assessment of publication bias was not performed because the included studies involved heterogeneous prediction objectives, outcome definitions, validation strategies, and performance metrics without a common effect estimate suitable for quantitative synthesis. Fifth, independent external validation remained rare, being reported in only one of the 26 included studies. Therefore, most performance estimates reflect development or internal validation settings, and the transportability of these models across populations, healthcare systems, and primary care workflows remains uncertain. Sixth, the primary evidence synthesis comprised 23 studies addressing current/prevalent prediabetes identification, whereas three longitudinal/prognostic studies were retained only as supplementary evidence. These prediction tasks differ in temporal structure, outcome definition, intended clinical use, and validation requirements. Accordingly, the conclusions of this review regarding discrimination, transportability, and implementation readiness primarily apply to current/prevalent prediabetes identification and should not be extrapolated to longitudinal/prognostic prediction of future prediabetes, impaired fasting glucose, or related dysglycaemic progression. Seventh, although the original search covered eight English- and Chinese-language databases, the updated search conducted during peer-review revision from 2 April 2025 to 1 August 2026 was restricted to PubMed, Embase, and Web of Science Core Collection. Thus, only three of the eight databases (37.5%) included in the original search were updated, and none of the four Chinese databases originally searched—CNKI, Wanfang, VIP, and the Chinese Biomedical Literature Database—was covered during the update period (4/4, 100% not updated). This limitation is particularly relevant to the applicability of the review to Chinese primary care populations. Of the 26 included studies, 12 (46.2%) were conducted in China, with one additional multi-cohort study including a Chinese population; among the 23 studies contributing to the primary current/prevalent identification evidence base, 10 (43.5%) were conducted in China, with one additional multi-cohort study including Chinese participants. Chinese populations therefore constitute a substantial component of the current evidence base. However, recent studies published after April 2025 exclusively in Chinese-language or regionally indexed journals may be underrepresented because no Chinese database was included in the update. Such studies may differ from internationally indexed studies in population characteristics, laboratory practices, predictor availability, modelling strategies, and healthcare workflows. Their underrepresentation may therefore affect the completeness of the evidence base and reduce confidence in conclusions regarding model applicability, transportability, and generalisability to contemporary Chinese primary care settings. The number of potentially eligible studies missed through this restricted update cannot be reliably quantified without rerunning the four Chinese databases over the same period; therefore, no numerical estimate of missed studies was inferred. Finally, subgroup fairness and real-world implementation were insufficiently evaluated in the primary literature. Most studies reported overall model performance without detailed assessment across socio-economic, ethnic, sex, age, or geographic subgroups. The absence of standardised fairness frameworks and subgroup reporting criteria further limited our ability to quantitatively compare equity-related performance across studies. Future studies should adopt standardised algorithmic fairness assessments and report specific fairness indicators, including demographic parity, equalised odds, and subgroup calibration, to determine whether model performance is consistent and equitable across clinically relevant population groups. Evidence regarding workflow integration, clinician and patient acceptance, resource requirements, cost-effectiveness, model updating, and long-term maintenance was also limited. These gaps restrict conclusions about equitable and sustainable implementation in routine primary care.

## 5 Conclusion

Machine learning-based models demonstrate potential to support current/prevalent prediabetes identification, particularly when incorporating routinely collected demographic, clinical, anthropometric, and lifestyle variables available in primary care settings. The primary evidence base comprised 23 studies evaluating current/prevalent prediabetes identification; three longitudinal/prognostic studies were retained only as supplementary evidence and were not used to support conclusions regarding the performance or implementation readiness of current/prevalent identification models. Many current/prevalent identification models showed promising discrimination performance; however, high discrimination observed during model development or internal validation does not establish readiness for routine clinical implementation. Independent external validation was reported in only one study, and nearly half of the included studies were judged to have an overall high risk of bias. Furthermore, evidence regarding calibration, clinical utility, subgroup fairness, and integration into real-world primary care workflows remains limited. Therefore, current evidence does not yet support routine deployment in primary care. Before a machine learning model can be considered ready for routine primary care use, it should demonstrate reproducible performance across multiple independent external populations, adequate calibration in the intended target population, clinically meaningful net benefit across pre-specified decision thresholds, successful prospective evaluation within real-world primary care workflows, and acceptable cost-effectiveness compared with existing screening strategies. As a pragmatic benchmark, validation across at least three independent populations may provide stronger evidence of transportability, although this should not be interpreted as a universal requirement. Calibration should be assessed using calibration plots and quantitative measures such as the calibration slope (ideal value 1) and calibration-in-the-large (ideal value 0), with interpretation in the intended target population and clinical context. Models should also demonstrate acceptable subgroup performance and fairness across relevant populations and be developed and validated using transparent, leakage-resistant procedures. These conclusions apply to current/prevalent prediabetes identification and should not be extrapolated to longitudinal/prognostic prediction of future prediabetes, impaired fasting glucose, or related dysglycaemic progression.

## Data Availability

The original contributions presented in the study are included in the article/Supplementary Material, further inquiries can be directed to the corresponding author.
