# Supplementary material for: Machine learning models for identifying prediabetes: a systematic review of performance, validation, and implications for primary care
Source: Front Med (Lausanne). 2026 Sep 28;13:1956800. doi: 10.3389/fmed.2026.1956800 (PMC13655083; doi:10.3389/fmed.2026.1956800)
Supplement: Supplementary file 1 [file Table1.docx]

Supplementary Material 1

# Complete database search strategies

Machine learning models for identifying prediabetes: a systematic review of performance, validation, and implications for primary care

Original searches were conducted from database inception to 1 April 2025. Strategies combined controlled vocabulary and free-text terms for machine learning/artificial intelligence, prediction modelling/risk assessment, and prediabetes. In response to peer review, updated searches of PubMed, Embase, and Web of Science Core Collection were conducted from 2 April 2025 to 1 August 2026 using the same database-specific search strategies as in the original search, with only the date limits extended. The updated searches identified 278 records before deduplication (PubMed, n=137; Embase, n=101; Web of Science Core Collection, n=40).

## PubMed

((machine learning[MeSH Terms] OR artificial intelligence[MeSH Terms] OR automated pattern recognition[MeSH Terms] OR regression analysis[MeSH Terms] OR deep learning[Title/Abstract] OR neural networks[Title/Abstract] OR AI[Title/Abstract] OR ML[Title/Abstract] OR classification[Title/Abstract] OR pattern recognition[Title/Abstract]) AND (risk assessment[MeSH Terms] OR predictive modeling[Title/Abstract] OR predictive model[Title/Abstract] OR prediction[Title/Abstract] OR forecasting[Title/Abstract] OR risk prediction[Title/Abstract] OR model[Title/Abstract] OR models[Title/Abstract]) AND (Prediabetic State[MeSH Terms] OR Prediabetic States[Title/Abstract] OR State, Prediabetic[Title/Abstract] OR States, Prediabetic[Title/Abstract] OR Prediabetes[Title/Abstract]))

## Embase

('machine learning'/exp OR 'artificial intelligence'/exp OR 'deep learning':ti,ab OR 'neural network*':ti,ab OR AI:ti,ab OR ML:ti,ab OR classification:ti,ab OR 'pattern recognition':ti,ab) AND ('risk assessment'/exp OR 'prediction model*':ti,ab OR 'predictive model*':ti,ab OR prediction:ti,ab OR forecasting:ti,ab OR 'risk prediction':ti,ab OR model*:ti,ab) AND ('prediabetes'/exp OR prediabetes:ti,ab OR 'prediabetic state*':ti,ab OR 'impaired fasting glucose':ti,ab OR 'impaired glucose tolerance':ti,ab)

## Web of Science Core Collection

TS=(("machine learning" OR "artificial intelligence" OR "deep learning" OR "neural network*" OR AI OR ML OR classification OR "pattern recognition") AND ("risk assessment" OR "predictive model*" OR "prediction model*" OR prediction OR forecasting OR "risk prediction" OR model*) AND (prediabetes OR "prediabetic state*" OR "impaired fasting glucose" OR "impaired glucose tolerance"))

## Cochrane Library

([mh "machine learning"] OR [mh "artificial intelligence"] OR "deep learning":ti,ab,kw OR "neural network*":ti,ab,kw OR AI:ti,ab,kw OR ML:ti,ab,kw OR classification:ti,ab,kw OR "pattern recognition":ti,ab,kw) AND ([mh "risk assessment"] OR "predictive model*":ti,ab,kw OR "prediction model*":ti,ab,kw OR prediction:ti,ab,kw OR forecasting:ti,ab,kw OR "risk prediction":ti,ab,kw OR model*:ti,ab,kw) AND ([mh "Prediabetic State"] OR prediabetes:ti,ab,kw OR "prediabetic state*":ti,ab,kw OR "impaired fasting glucose":ti,ab,kw OR "impaired glucose tolerance":ti,ab,kw)

## CNKI

主题=(机器学习 OR 人工智能 OR 深度学习 OR 神经网络 OR 分类 OR 模式识别) AND 主题=(预测模型 OR 风险预测 OR 预测 OR 风险评估 OR 模型) AND 主题=(糖尿病前期 OR 糖调节受损 OR 空腹血糖受损 OR 糖耐量受损)

## CBM

主题词/关键词=(机器学习 OR 人工智能 OR 深度学习 OR 神经网络 OR 分类 OR 模式识别) AND (预测模型 OR 风险预测 OR 预测 OR 风险评估 OR 模型) AND (糖尿病前期 OR 糖调节受损 OR 空腹血糖受损 OR 糖耐量受损)

## VIP

题名或关键词=(机器学习 OR 人工智能 OR 深度学习 OR 神经网络 OR 分类 OR 模式识别) AND (预测模型 OR 风险预测 OR 预测 OR 风险评估 OR 模型) AND (糖尿病前期 OR 糖调节受损 OR 空腹血糖受损 OR 糖耐量受损)

## Wanfang

主题=(机器学习 OR 人工智能 OR 深度学习 OR 神经网络 OR 分类 OR 模式识别) AND 主题=(预测模型 OR 风险预测 OR 预测 OR 风险评估 OR 模型) AND 主题=(糖尿病前期 OR 糖调节受损 OR 空腹血糖受损 OR 糖耐量受损)

# Updated search conducted during peer-review revision

The updated search was conducted in PubMed, Embase, and Web of Science Core Collection for records published from 2 April 2025 to 1 August 2026. The same database-specific search strategies used in the original review were retained, and only the date limits were extended. The same eligibility criteria were applied to records identified through the updated search. The Cochrane Library, CNKI, CBM, VIP, and Wanfang searches were not rerun during the peer-review update.

## PubMed

**Search strategy:** ((machine learning[MeSH Terms] OR artificial intelligence[MeSH Terms] OR automated pattern recognition[MeSH Terms] OR regression analysis[MeSH Terms] OR deep learning[Title/Abstract] OR neural networks[Title/Abstract] OR AI[Title/Abstract] OR ML[Title/Abstract] OR classification[Title/Abstract] OR pattern recognition[Title/Abstract]) AND (risk assessment[MeSH Terms] OR predictive modeling[Title/Abstract] OR predictive model[Title/Abstract] OR prediction[Title/Abstract] OR forecasting[Title/Abstract] OR risk prediction[Title/Abstract] OR model[Title/Abstract] OR models[Title/Abstract]) AND (Prediabetic State[MeSH Terms] OR Prediabetic States[Title/Abstract] OR State, Prediabetic[Title/Abstract] OR States, Prediabetic[Title/Abstract] OR Prediabetes[Title/Abstract]))

**Date limit:** 2 April 2025 to 1 August 2026

**Records retrieved:** 137

## Embase

**Search strategy:** ('machine learning'/exp OR 'artificial intelligence'/exp OR 'deep learning':ti,ab OR 'neural network*':ti,ab OR AI:ti,ab OR ML:ti,ab OR classification:ti,ab OR 'pattern recognition':ti,ab) AND ('risk assessment'/exp OR 'prediction model*':ti,ab OR 'predictive model*':ti,ab OR prediction:ti,ab OR forecasting:ti,ab OR 'risk prediction':ti,ab OR model*:ti,ab) AND ('prediabetes'/exp OR prediabetes:ti,ab OR 'prediabetic state*':ti,ab OR 'impaired fasting glucose':ti,ab OR 'impaired glucose tolerance':ti,ab)

**Date limit:** 2 April 2025 to 1 August 2026

**Records retrieved:** 101

## Web of Science Core Collection

**Search strategy:** TS=(("machine learning" OR "artificial intelligence" OR "deep learning" OR "neural network*" OR AI OR ML OR classification OR "pattern recognition") AND ("risk assessment" OR "predictive model*" OR "prediction model*" OR prediction OR forecasting OR "risk prediction" OR model*) AND (prediabetes OR "prediabetic state*" OR "impaired fasting glucose" OR "impaired glucose tolerance"))

**Date limit:** 2 April 2025 to 1 August 2026

**Records retrieved:** 40

**Total records retrieved from the updated searches before deduplication:** 278.
