# Supplementary material for: Machine learning models for identifying prediabetes: a systematic review of performance, validation, and implications for primary care
Source: Front Med (Lausanne). 2026 Sep 28;13:1956800. doi: 10.3389/fmed.2026.1956800 (PMC13655083; doi:10.3389/fmed.2026.1956800)
Supplement: Supplementary file 2 [file Table2.docx]

PRISMA 2020 checklist

Machine learning models for identifying prediabetes: a systematic review of performance, validation, and implications for primary care

Completed checklist for submission to Frontiers in Medicine, Family Medicine and Primary Care.

| **Section/topic** | **Item** | **PRISMA 2020 checklist item** | **Location in submitted materials** |
| --- | --- | --- | --- |
| Title | 1 | Identify the report as a systematic review. | Title and first page |
| Abstract | 2 | Provide a structured abstract. | Abstract (structured single paragraph) |
| Rationale | 3 | Describe the rationale for the review in the context of existing knowledge. | Section 1, Introduction |
| Objectives | 4 | Provide an explicit statement of the objective(s) or question(s) the review addresses. | Section 1, Introduction (final paragraph) |
| Eligibility criteria | 5 | Specify the inclusion and exclusion criteria for the review. | Section 2.2, Review question and eligibility criteria |
| Information sources | 6 | Specify all databases and other sources searched, with date of last search. | Section 2.3, Information sources and search strategy |
| Search strategy | 7 | Present the full search strategies for all databases, registers and websites. | Supplementary Material 1, Complete database search strategies |
| Selection process | 8 | Specify the methods used to decide whether a study met inclusion criteria. | Section 2.4, Study selection and data extraction |
| Data collection process | 9 | Specify the methods used to collect data from reports. | Section 2.4, Study selection and data extraction |
| Data items | 10a/10b | List and define outcomes and other variables for which data were sought. | Sections 2.2 and 2.4 |
| Study risk of bias assessment | 11 | Specify methods used to assess risk of bias in included studies. | Section 2.5, Risk of bias and applicability |
| Effect measures | 12 | Specify effect measures or performance measures. | Sections 2.2 and 2.4; Section 3 |
| Synthesis methods | 13 | Describe processes used to prepare data and synthesize results. | Section 2.6, Data synthesis |
| Reporting bias assessment | 14 | Describe methods used to assess risk of bias due to missing results. | Not assessed; descriptive synthesis was used and reporting bias was not evaluated statistically |
| Certainty assessment | 15 | Describe methods used to assess certainty in the body of evidence. | Not assessed; no GRADE or equivalent certainty assessment was performed |
| Study selection | 16a/16b | Describe results of search and selection process, ideally using a flow diagram. | Section 3.1, Study selection; Figure 1 |
| Study characteristics | 17 | Cite each included study and present its characteristics. | Section 3.2, Study characteristics; Table 1 |
| Risk of bias in studies | 18 | Present assessments of risk of bias for each included study. | Section 3.6, Risk of bias and applicability; Supplementary Material 2, Table S3 |
| Results of individual studies | 19 | Present results for all outcomes for each study. | Sections 3.2–3.6; Supplementary Material 2, Tables S1–S3 |
| Results of syntheses | 20a-20d | Present results of each synthesis conducted. | Sections 3.2–3.6 |
| Reporting biases | 21 | Present assessments of risk of bias due to missing results. | Not assessed; descriptive synthesis was used and reporting bias was not evaluated statistically |
| Certainty of evidence | 22 | Present assessments of certainty in the body of evidence. | Not assessed; no GRADE or equivalent certainty assessment was performed |
| Discussion | 23a-23d | Discuss results, limitations of evidence, limitations of review processes, and implications. | Section 4, Discussion |
| Registration and protocol | 24a-24c | Provide registration information and explain any amendments. | Abstract; Section 2.1, Study design and protocol |
| Support | 25 | Describe sources of financial or non-financial support. | Funding |
| Competing interests | 26 | Declare competing interests of review authors. | Conflict of interest |
| Availability of data, code and other materials | 27 | Report which data and materials are publicly available. | Data availability statement; Supplementary Materials 1 and 2 |
