# Supplementary material for: Machine learning models for identifying prediabetes: a systematic review of performance, validation, and implications for primary care
Source: Front Med (Lausanne). 2026 Sep 28;13:1956800. doi: 10.3389/fmed.2026.1956800 (PMC13655083; doi:10.3389/fmed.2026.1956800)
Supplement: Supplementary file 3 [file Table3.docx]

Supplementary Material 2

# Supplementary tables

Machine learning models for identifying prediabetes: a systematic review of performance, validation, and implications for primary care

This file contains detailed information on predictors, model development and performance, PROBAST risk-of-bias and applicability assessments, predictor–reference-standard overlap, the ML-specific methodological audit, and sensitivity analyses.

Table S1. Predictors used in the included models

| **First author** | **Candidate or retained predictors** | **Number of predictors** | **Most important predictors** | **Predictor selection method** | **Missing-data handling** |
| --- | --- | --- | --- | --- | --- |
| Xiaolong Li(37) | Age, TG, TC, BMI, Apolipoprotein B, TP, leukocyte count, HDL-C, hypertension, ALT, d3protn, UA, LDL_C, FET, d3carbo, d3kcal, Income | 17 | Age, TG, TC, BMI, ApolipoproteinB, TP, leukocyte count, HDL-C, and hypertension. | LASSO regression | Imputation Method |
| Qianqian Yang(38) | Age, BMI, Diastolic Blood Pressure, Systolic Blood Pressure, Triglycerides, HDL-C, Aspartate/Alanine, Creatinine, UA, Urinary Glucose, Urinary Protein | 11 | Age, Triglycerides, Aspartate/Alanine, Systolic Blood Pressure, BMI | LASSO regression | — |
| Xiaodong Zhang(36) | Age, BMI, ALT, ALT/AST Ratio, Alkaline Phosphatase, Total Bilirubin, Direct Bilirubin, Globulin, Albumin/Globulin, Creatinine, UA, Blood Urea Nitrogen/Creatinine, Triglycerides, LDL-C, Fasting Glucose, Lymphocyte Count, Monocyte Count, Hemoglobin, RBC Count, Platelet Count, Triglyceride-Glucose Index | 21 | Age, Fasting Glucose, Hemoglobin, RBC Count, BMI, Triglyceride-Glucose Index | LASSO regression | Deletion Method |
| Hung Viet Nguyen(35) | Age, Personal Income Quintile, Total Household Income, Hypertension Diagnosis Time, DI1_pt, Use of Antihypertensive Drugs, DI2_pt, DI2_2, Alcohol Consumption Frequency in the Past Year, Alcohol-Induced Harm to Others, Smoking, Physical Activity Time, Moderate Intensity Physical Activity Time, Fasting Time, First Systolic Pressure, First Diastolic Pressure, Second Systolic Pressure, Third Systolic Pressure, Kidney Height, Waist Circumference, Neck Circumference, BMI, Triglycerides, AST, ALT, Energy Intake, Carbohydrate Intake, Platelet Count | 38 | Age, BMI, Neck Circumference, ALT, Leukocyte Count, Daily Energy Intake, Carbohydrate Intake, Third Systolic Pressure, Personal Income Quintile, Platelet Count | Minimum Redundancy Maximum Relevance (mRMR) | Deletion Method |
| Myoung-Lyun Heo(33) | Age, Thiamine Intake, Water Intake, Gender, Education Level, Food Intake, Marital Status, Calcium Intake, Occupation, Polyunsaturated Fatty Acid Intake | 10 | Age, Thiamine Intake, Water Intake | Based on Gini Coefficient | Exclusion Method |
| Katherine Lu(34) | Age, BMI, Total Cholesterol, Depression Status, Hypertension Status, Osteoporosis, Chronic Obstructive Pulmonary Disease, Steroid Use, Antihypertensive Drug Use, Gender | 10 | Age, BMI, Antihypertensive Drug Use | Literature Review, Data Completeness, Collinearity Evaluation | Deletion Method |
| Qiyuan Cui(32) | Systolic Blood Pressure, Fatty Liver, BMI, Waist Circumference, Triglycerides, Serum Creatinine, Age, Diastolic Blood Pressure, Apolipoprotein B, Total Cholesterol, Serum Uric Acid, Gamma-Glutamyl Transferase | 12 | Systolic Blood Pressure, Fatty Liver, Waist Circumference, Serum Creatinine | Multivariate Logistic Regression Analysis | Imputation Method |
| Xiaohong Chen(31) | Age, Gender, Weight, Height, BMI, Diastolic Blood Pressure, Systolic Blood Pressure, Urinary Protein, Total Protein, Globulin, Total Bilirubin, Direct Bilirubin, Indirect Bilirubin, ALT, AST, Blood Urea Nitrogen, Uric Acid, Total Cholesterol, Triglycerides, HDL-C | 20 | Age, Urinary Protein, Total Protein, ALT | Univariate Analysis, Multivariate Logistic Regression Analysis | Deletion Method |
| Oana VÎRGOLICI(30) | Gender, Blood Glucose, Triglycerides, HDL-C, Waist Circumference, Systolic Blood Pressure | 6 | Gender, Blood Glucose, Triglycerides, HDL-C, Waist Circumference, Systolic Blood Pressure | Expert Knowledge, Literature | No Missing Data |
| Qianying Ou(29) | Gender, Age, Education Level, Family Income, Salt Intake, Smoking Status, Family History of Diabetes, Hypertension, Hyperlipidemia, Thyroid Disease, Complications, BMI, Waist Circumference, Triglycerides, Steroid Use, Total Cholesterol, LDL-C, HDL-C, Uric Acid, TSH, Thyroid Peroxidase Antibody, Antithyroglobulin Antibody, Urinary Iodine, Vitamin D3, Fasting Insulin, 2-hour Postprandial Insulin, Fasting Insulin, 2-hour Postprandial Insulin | 28 | Age, Complications, Education, Family History of Diabetes, LDL-C, HDL-C, Uric Acid, Waist Circumference, Hypertension, Triglycerides, TSH, Antithyroglobulin Antibody, 2-hour Postprandial Insulin, and 2-hour Postprandial Insulin Precursor | LASSO Regression | — |
| Yanhua Hu(28) | BMI, Systolic Blood Pressure, Age, HDL-C, Gender, Serum Creatinine, Total Cholesterol, ALT, Diastolic Blood Pressure, Family History of Diabetes, Triglycerides, LDL-C, Blood Urea Nitrogen, Fasting Glucose, AST, Alcohol Consumption, Smoking Status | 17 | Age, Fasting Glucose, BMI, Serum Creatinine, Systolic Blood Pressure, Triglycerides | LASSO Regression | — |
| Di Gong(27) | Glucose Metabolism Status, Age, Gender, BMI, Systolic Blood Pressure, Urinary Glucose, Urinary Protein, Total Protein, Albumin, Globulin, Total Bilirubin, Direct Bilirubin, Indirect Bilirubin, ALT, AST, Blood Urea Nitrogen, Serum Creatinine, Uric Acid, Total Cholesterol, Triglycerides, LDL-C, HDL-C | 22 | Age, BMI, Systolic Blood Pressure, Urinary Glucose, Urinary Protein, Total Protein, Globulin, ALT, HDL-C | Literature, Univariate Analysis, Multivariate Logistic Regression Analysis | Exclusion Method |
| Savitesh Kushwaha(26) | Age, Gender, Weight, Height, Triceps Skinfold Thickness, Scapular Skinfold Thickness, Mid-Upper Arm Circumference, Waist Circumference | 8 | Triceps Skinfold Thickness, Mid-Upper Arm Circumference, Waist Circumference, Age, Height | Literature | Imputation Method |
| Weinan Dong(25) | Age, BMI, Waist-Hip Ratio, Systolic Blood Pressure, Waist Circumference, Smoking Status, Sleep Duration, Weekly Intense Leisure Activity Time, Weekly Fruit Intake | 8 | Age, BMI | Multivariate Logistic Regression Analysis | No Missing Data |
| Jun Li(24) | Tongue Body Color Features, Texture Features, Depth Features | 34 | b-Channel in the Lab Color Space, Tongue Coating Saturation | Feature Extraction, Fusion | — |
| Mostafa Abbas(23) | Age, Gender, BMI, Waist Circumference, Hip Circumference, Systolic Blood Pressure, Diastolic Blood Pressure, Pulse, Smoking, Waterpipe Smoking, Other Family Smokers, Snoring, Occupation | 13 | Age, Gender, BMI, Waist Circumference, Blood Pressure | Multivariate Logistic Regression Analysis | Imputation Method |
| Liyang Wang(22) | 12-lead ECG, 12-lead ECG used for 5 seconds | 1 | 12-lead ECG, 12-lead ECG used for 5 seconds | — | Exclusion Method |
| E Hai Wang(21) | Age, Waist Circumference, Smoking History, Fatty Liver, Fasting Glucose, HbA1c | 6 | Age, Waist Circumference, Smoking History, Fatty Liver, Fasting Glucose, HbA1c | Univariate Analysis, Multivariate Analysis | — |
| Soo Beom Choi(20) | Age, Gender, Family History of Diabetes, Alcohol Consumption, BMI, Waist Circumference, Hypertension | 7 | Age, Gender, Family History of Diabetes, Alcohol Consumption, BMI, Waist Circumference, Hypertension | Multivariate Analysis | — |
| Aarthi Yesupatham(39) | Age, gender, total antioxidant status (TAS), HbA1c, fasting blood glucose (FBG), oral glucose tolerance test (OGTT), triglycerides, HDL, LDL, VLDL, total cholesterol, waist circumference, hemoglobin, BMI | 14 | BMI, waist circumference, age, TAS, HbA1c, FBG, OGTT | Pearson correlation and feature-importance analysis; no formal dimension-reduction procedure reported | Missing values were reported as handled during preprocessing, but the specific method was not described |
| Maher Maalouf(40) | Lipids (TG, TC, HDL, LDL), oxidative-stress markers (GSH, GSSG, GSH/GSSG, 8-OHdG), mitochondrial markers (Humanin, MOTS-c, p66Shc), inflammatory markers (CRP, IL-6, IL-1β, IL-10, MCP-1, IGF-1), HbA1c, age | 19 | IGF-1, IL-10, CRP in the best inflammatory-only panel | VIF-based collinearity reduction within training folds; biomarker panel and model selected by nested-CV AUC | Complete analytic dataset reported; no imputation described for the final analytic data |
| Lin Wang(41) | Age, FPG, high blood pressure, BMI, waist-to-hip ratio, total cholesterol, triglycerides, HDL-C, LDL-C | 9 | FPG, HDL-C, total cholesterol, high blood pressure, BMI, waist-to-hip ratio | Predefined clinical variables; SHAP and model-based feature importance used for interpretation | Listwise deletion of observations with any missing value |
| Krithi Pushpanathan(42) | Macula-centred retinal fundus photographs | 1 | Image-derived retinal features; saliency maps predominantly highlighted retinal vasculature | End-to-end ViT/ResNet-50 representation learning; Grad-CAM used for interpretation | Poor-quality/ungradable images were excluded; no tabular imputation applicable |
| Huaxin Fan(43) | Gender, age, urea, creatinine, HbA1c, cholesterol, triglycerides, HDL, LDL, VLDL, BMI | 11 | Gender, age, HbA1c, cholesterol, triglycerides, BMI | Multiclass LASSO performed on training data only | No imputation method clearly reported; exact duplicates were removed and format inconsistencies corrected |
| Marwa Matboli(44) | 11 molecular markers (miR-342, miR-636, miR-15b, miR-611, RET, IGF1R, mTOR, HSPA1B, DDX58, NFKB1, RB1CC1) and 14 biochemical/clinical markers (fasting glucose, postprandial glucose, HbA1c, insulin, HOMA-IR, total cholesterol, systolic and diastolic blood pressure, LDL-C, HDL-C, triglycerides, albumin/creatinine ratio, creatinine, eGFR) | 25 | RFECV-retained combined features: miR-342, miR-636, miR-15b, NFKB1, FastingGlucose, HDL-C, albumin/creatinine ratio; top five by feature importance: miR-342, NFKB1, miR-636, albumin/creatinine ratio, HDL-C | RFECV with RandomForestClassifier and feature-importance analysis | No missing values reported |
| Junho Cha(45) | Age, sex, BMI, HDL cholesterol, 39 selected metabolites, and 86 selected SNPs in the integrated model | 129 | Integrated clinical-metabolomic-genomic panel; no single dominant predictor reported for the final model | Full-dataset association/FDR screening for metabolites; GWAS p-value screening and LD pruning for SNPs before predictive modelling | Participants with missing genotype, metabolite, or clinical data were excluded |

Abbreviations: ALT, alanine aminotransferase; AST, aspartate aminotransferase; BMI, body mass index; CRP, C-reactive protein; eGFR, estimated glomerular filtration rate; FBG/FPG, fasting blood/plasma glucose; HbA1c, glycated haemoglobin; HDL-C, high-density lipoprotein cholesterol; HOMA-IR, homeostatic model assessment of insulin resistance; IGF-1, insulin-like growth factor 1; IL, interleukin; LASSO, least absolute shrinkage and selection operator; LD, linkage disequilibrium; LDL-C, low-density lipoprotein cholesterol; MCP-1, monocyte chemoattractant protein 1; mRMR, minimum redundancy maximum relevance; OGTT, oral glucose tolerance test; RBC, red blood cell; RFECV, recursive feature elimination with cross-validation; SNP, single-nucleotide polymorphism; TAS, total antioxidant status; TC, total cholesterol; TG, triglycerides; TP, total protein; TSH, thyroid-stimulating hormone; UA, uric acid; VIF, variance inflation factor; VLDL, very-low-density lipoprotein. Dataset-specific variable labels are retained as reported by the original studies. A dash indicates that the information was not reported.

Table S2. Model development, validation and reported performance

| **First author** | **Modelling methods** | **Validation method** | **Best-performing model** | **AUC (development/internal validation)** | **Sensitivity** | **Specificity** | **Accuracy** | **External-validation AUC** | **Clinical utility assessment** |
| --- | --- | --- | --- | --- | --- | --- | --- | --- | --- |
| Xiaolong Li(37) | XGBoost, RF, SVM, NB, ANN, DT, LR | Random Split, 5-Fold Cross Validation | XGBoost | 0.978/0.939 | 0.896 | 0.841 | 0.845 | — | Not reported |
| Qianqian Yang(38) | LR, RF, SVM, XGBoost, ANN | Random Split | XGBoost | —/0.8288 | 0.8098 | 0.6925 | 0.7513 | — | Not reported |
| Xiaodong Zhang(36) | LR, RF, SVM, Extreme Gradient Boosting, Multilayer Perceptron, LightGBM, CatBoost | Random Split ,10-Fold Cross Validation | CatBoost | —/0.806 | 0.746 | 0.716 | 0.73 | — | DCA performed; clinical benefit reported for CatBoost |
| Hung Viet Nguyen(35) | TabPFN, HyperTab, LightGBM, XGBoost, Gradient Boosting, AdaBoost, RF, LR | Random Split | Hybrid Model  HyperTab-LIME | —/0.8429 | 0.7632 | 0.7686 | 0.7565 | — | Not reported |
| Myoung-Lyun Heo(33) | LR, kNN, RF | Random Split, 5-Fold Cross Validation | RF | 0.61/0.49 | 0.84 | — | 0.71 | — | Not reported |
| Katherine Lu(34) | LR, RF, XGBoost, Hybrid Naive Bayes, KNN, SVM, DNN | Random Split | DNN Model | —/0.76 | 0.6 | 0.81 | 0.69 | — | Not reported |
| Qiyuan Cui(32) | XGBoost, SVM, NB, DT, LR | Random Split,5-Fold Cross Validation | XGBoost | 0.8264/0.7391 | 0.7561 | 0.6245 | 0.6763 | — | DCA performed; net benefit reported for XGBoost |
| Xiaohong Chen(31) | LR, RF, SVM, XGBoost | Random Split ,5-Fold Cross Validation | LR | 0.78/0.78 | 0.76 | 0.5 | 0.75 | — | Not reported |
| Oana VÎRGOLICI(30) | Multilayer Perceptron Neural Network | Random Split | Multilayer Perceptron Neural Network | — | — | — | 0.96 | — | Not reported |
| Qianying Ou(29) | LR, SVM, RF, LDA, NB, Treebag | Random Split,10-Fold Cross Validation | LASSO Model | —/0.695 | 0.044 | 0.995 | 0.74 | — | Not reported |
| Yanhua Hu(28) | Full Cox Proportional Hazard Model, Stepwise Cox Proportional Hazard Model, MFP, LASSO | Random Split | LASSO Model | 0.7341/0.7336 | 0.653 | 0.699 | — | — | DCA performed in training and validation cohorts; net benefit assessed |
| Di Gong(27) | Full Variable Prediction Model, Simplified Prediction Model | Random Split | Full Variable Prediction Model, Simplified Prediction Model | Full.model:0.81/0.82  Simplified.model:0.8/0.81 | 0.91/0.91 | — | 0.79/0.77 | — | Not reported |
| Savitesh Kushwaha(26) | GBM, RF, Extra Trees Classifier, DT, SVM, XGBoost | Random Split, 10-Fold Cross Validation | XGBoost | —/0.959 | 0.89 | — | 0.942 | — | Not reported |
| Weinan Dong(25) | LR, XGBoost | Random Split,5-Fold Cross Validation | XGBoost | 0.822/0.822 | 0.724 | 0.779 | — | — | Not reported |
| Jun Li(24) | XGBoost | Random Split ,10-Fold Cross Validation | XGBoost | 0.924/0.918 | 0.81 | — | 0.821 | — | Not reported |
| Mostafa Abbas(23) | LR, RF, GBM, XGBoost, DL | Random Split,10-Fold Cross Validation | LR Model | 0.79/0.80 | 0.86 | 0.579 | 0.67 | — | Not reported |
| Liyang Wang(22) | Convolutional Neural Network | 5-Fold Cross Validation | IGRNet Model | 0.809/0.773 | 0.862 | 0.865 | 0.854 | — | Not reported |
| E Hai Wang(21) | Multivariable Cox Regression Analysis | Random Split | Model 2 | 0.87/0.87 | 0.87 | 0.73 | — | — | Not reported |
| Soo Beom Choi(20) | ANN, SVM, LR | Random Split,10-Fold Cross Validation | SVM Model | —/0.761 | 0.789 | 0.612 | 0.649 | — | Not reported |
| Aarthi Yesupatham(39) | PNN, SVM, KNN, LR | Random split (70% training, 15% validation, 15% test); 10-fold CV on the training set | PNN | 0.9808 (internal test) | 0.96154 | — | 0.98333 | — | Not reported |
| Maher Maalouf(40) | LR, RF, LightGBM, XGBoost | 80/20 development-holdout split; nested CV (outer 5-fold, inner 3-fold); calibrated internal holdout evaluation | Inflammatory-only LightGBM | 0.743 (nested CV); 0.711 (internal holdout) | 0.500 | 0.877 | 0.780 | — | Not reported |
| Lin Wang(41) | SVM, DT, RF, LR, KNN, NB, AdaBoost, GBM | Random split 7:3 (training n=781; validation n=336) | RF (best overall metrics); LR had highest AUC | RF 0.9558; LR 0.9731 | 0.914 (RF) | — | 0.967 (RF) | — | Not reported |
| Krithi Pushpanathan(42) | ViT-based model with ResNet-50 patch projection | Individual-specific 70/15/15 internal split in SEED+UKBB; independent external validation in SP2 and BES | ViT-ResNet-50 | 0.746 (95% CI 0.733–0.759) | 0.685 | 0.669 | — | SP2: 0.590 (0.569–0.611); BES: 0.523 (0.488–0.557) | Not reported |
| Huaxin Fan(43) | Multinomial LR, SVM, RF, XGBoost, basic MLP, rule-based DT, ProgMDD | Stratified 70/10/20 split plus stratified 5-fold CV; preprocessing and SMOTE-Tomek restricted to training data | ProgMDD | 0.9913 (Prediabetes, held-out test) | 0.88 (Prediabetes recall) | — | 0.9759 (overall holdout) | — | Not reported |
| Marwa Matboli(44) | RF, Extra Trees, QDA, NB, LightGBM | 70/30 train-test split; stratified 5-fold CV; SMOTE within training folds | Extra Trees (combined model) | 0.9989 (overall multiclass test AUC) | 1.00 (Prediabetes recall) | — | 0.9744 (overall) | — | Not reported |
| Junho Cha(45) | Baseline-category LR: Clinical, Metabolite-Enriched, Integrated Multi-omics models | Stratified 5-fold CV | Integrated Multi-omics model | 0.935 (overall multiclass CV AUC) | 0.888 (overall) | 0.525 (overall) | 0.646 (overall) | — | Not reported |

Abbreviations: DCA, decision-curve analysis; ANN, artificial neural network; AUC, area under the receiver operating characteristic curve; CatBoost, categorical boosting; CI, confidence interval; CV, cross-validation; DNN, deep neural network; DT, decision tree; GBM, gradient boosting machine; IGRNet, impaired glucose regulation network; k-NN/KNN, k-nearest neighbours; LASSO, least absolute shrinkage and selection operator; LDA, linear discriminant analysis; LightGBM, light gradient boosting machine; LIME, local interpretable model-agnostic explanations; LR, logistic regression; MFP, multivariable fractional polynomial; ML, machine learning; MLP, multilayer perceptron; NB, naive Bayes; PNN, pattern neural network; QDA, quadratic discriminant analysis; RF, random forest; SVM, support vector machine; TabPFN, tabular prior-data fitted network; ViT, vision transformer; XGBoost, extreme gradient boosting. Values are reproduced as reported by the included studies; a dash indicates that the metric was not reported. Independent external-validation AUCs are reported separately from development/internal-validation estimates. Internal holdout sets and cross-validation are not classified as external validation. Generic descriptions of modelling approaches (e.g., “machine learning”) without a clearly specified algorithm were not counted as independent models. When multiple algorithms were evaluated within a study, the model with the best reported performance according to the original study was extracted for summary.

Table S3. PROBAST risk-of-bias and applicability assessment

| **Study** | **Risk of bias** | | | | **Applicability concerns** | | | **Overall judgement** | |
| --- | --- | --- | --- | --- | --- | --- | --- | --- | --- |
| **First author** | **Participants** | **Predictors** | **Outcome** | **Analysis** | **Participants** | **Predictors** | **Outcome** | **Risk of bias** | **Applicability concerns** |
| Xiaolong Li(37) | Low | Low | Low | Low | Low | Low | Low | Low | Low |
| Qianqian Yang(38) | High | High | High | High | High | High | High | High | High |
| Xiaodong Zhang(36) | Low | Low | Low | Low | Low | Low | Low | Low | Low |
| Hung Viet Nguyen(35) | Low | Low | Low | Low | Low | Low | Low | Low | Low |
| Myoung-Lyun Heo(33) | Low | Low | Low | Low | Low | Low | Low | Low | Low |
| Katherine Lu(34) | Low | Low | Low | Low | Low | Low | Low | Low | Low |
| Qiyuan Cui(32) | Low | Low | Low | Low | Low | Low | Low | Low | Low |
| Xiaohong Chen(31) | High | High | High | High | High | High | High | High | High |
| Oana VÎRGOLICI(30) | High | High | High | High | High | High | High | High | High |
| Qianying Ou(29) | High | High | High | High | High | High | High | High | High |
| Yanhua Hu(28) | Low | High | High | High | Low | Low | Low | High | Low |
| Di Gong(27) | High | High | High | High | High | Low | Low | High | High |
| Savitesh Kushwaha(26) | Low | Low | Low | Low | Low | Low | Low | Low | Low |
| Weinan Dong(25) | Low | High | High | High | Low | Low | Low | High | Low |
| Jun Li(24) | Low | High | High | High | Low | Low | Low | High | Low |
| Mostafa Abbas(23) | Low | Low | Low | Low | Low | Low | Low | Low | Low |
| Liyang Wang(22) | Low | Low | Low | Low | Low | Low | Low | Low | Low |
| E Hai Wang(21) | Low | Low | Low | Low | Low | Low | Low | Low | Low |
| Soo Beom Choi(20) | Low | Low | Low | Low | Low | Low | Low | Low | Low |
| Aarthi Yesupatham(39) | Low | High | High | High | Low | Low | Low | High | Low |
| Maher Maalouf(40) | Low | Low | Low | Low | Low | Low | Low | Low | Low |
| Lin Wang(41) | Low | Low | Low | Low | High | Low | High | Low | High |
| Krithi Pushpanathan(42) | Low | Low | Low | Low | Low | Low | Low | Low | Low |
| Huaxin Fan(43) | High | Low | High | High | High | Low | High | High | High |
| Marwa Matboli(44) | High | Low | High | High | High | Low | Low | High | High |
| Junho Cha(45) | Low | Low | Low | High | Low | Low | Low | High | Low |

PROBAST domains are presented using the standard terminology of participants, predictors, outcome and analysis. Risk of bias is classified as Low or High. Applicability is expressed as concern (Low or High); Applicability concerns were assessed according to PROBAST criteria and classified as low, high, or unclear concern across the three applicability domains (participants, predictors, and outcome).

Table S4. Study-level assessment of glycaemic predictor–reference-standard overlap and potential incorporation bias

| **Study** | **Prediabetes reference standard / outcome definition** | **Glycaemic predictor(s) used in model** | **Relationship between predictor and reference standard** | **Paired model without overlapping glycaemic marker available?** | **Quantitative AUC comparison relevant to overlap** | **Incorporation-bias judgement*** |
| --- | --- | --- | --- | --- | --- | --- |
| **Choi et al. (20)** | Prediabetes identified by fasting-glucose-based clinical criteria | None | No glycaemic predictor–reference-standard overlap identified | No | Not estimable | **No direct overlap identified** |
| **Wang et al. (21)** | Longitudinal glycaemic outcome assessed during follow-up using FBG/HbA1c-related criteria | Baseline FBG; HbA1c | Same types of glycaemic markers were used prognostically, but baseline predictors preceded subsequent outcome ascertainment | No | AUC 0.87 in development and 0.87 in validation; no paired model excluding FBG/HbA1c | **Temporally separated; not classified as direct contemporaneous incorporation** |
| **Wang et al. (22)** | Prediabetes/IGR defined using WHO IFG/IGT criteria and clinical diagnosis | None; ECG signal only | No glycaemic marker used as model input | No | Not applicable | **No direct overlap identified** |
| **Abbas et al. (23)** | Prediabetes defined using HbA1c-based criteria | None | Outcome defined by HbA1c, but HbA1c was not used as a model predictor | No | Not applicable | **No direct overlap identified** |
| **Li et al. (24)** | Prediabetes/diabetes status determined using clinical glycaemic classification | None; tongue-image features only | Model inputs were image-derived and did not include glycaemic diagnostic markers | No | Not applicable | **No direct overlap identified** |
| **Dong et al. (25)** | Prediabetes defined using fasting glucose and/or HbA1c criteria | None | Non-laboratory predictors only; diagnostic glycaemic variables were not model inputs | No | Not applicable | **No direct overlap identified** |
| **Kushwaha et al. (26)** | Normal/prediabetes grouping based on HbA1c-related classification | None | Non-invasive anthropometric predictors; HbA1c was not used as a predictor | No | Not applicable | **No direct overlap identified** |
| **Gong et al. (27)** | Glucose-metabolism categories based on FBG/OGTT-related criteria | Urinary glucose was included among retained variables; blood FBG/2-h glucose was not retained in the reported final prediabetes model | Related glycaemic information, but not the same blood-glucose measurement used in the reference standard | No | No paired otherwise-identical model reported | **Related but non-identical glycaemic information; no confirmed direct incorporation** |
| **Hu et al. (28)** | **Incident prediabetes during 5-year follow-up**, defined using follow-up fasting-glucose criteria | Baseline fasting glucose | Baseline FPG was measured before subsequent incident outcome ascertainment | No | Internal AUC 0.7336; no paired model without baseline FPG | **Temporally separated; not classified as direct contemporaneous incorporation** |
| **Ou et al. (29)** | Prediabetes defined using FPG, 2-h post-load glucose and/or HbA1c criteria | Fasting insulin, 2-h postprandial insulin/proinsulin-related measures; no FPG/HbA1c predictor | Glycaemic-related endocrine markers but not the diagnostic glucose/HbA1c measurements themselves | No | Not estimable | **No direct same-marker overlap identified** |
| **Vîrgolici et al. (30)** | Output defined by ATP III rule: ≥3 of 5 abnormal components, including serum glucose, TG, HDL-C, waist circumference and SBP | Serum glucose, TG, HDL-C, waist circumference and SBP, plus sex | The same variables used to construct the output label were entered directly as predictors | No | AUC not reported; reported test accuracy was 0.96 | **Direct predictor–reference-standard overlap: High risk of incorporation bias** |
| **Chen et al. (31)** | Prediabetes/glucose-metabolism classification based on FBG/OGTT-related criteria | No blood glucose or HbA1c retained among the reported predictors | Diagnostic glycaemic measurements were not used as final model predictors | No | Not applicable | **No direct overlap identified** |
| **Cui et al. (32)** | Development of incident IFG over approximately 5-year follow-up, using WHO fasting-glucose/OGTT criteria | No FPG/HbA1c in the final model; principal variables included SBP, fatty liver, waist circumference and serum creatinine | Outcome was longitudinal and final model did not use the defining glycaemic marker | No | Validation AUC 0.7391; no relevant paired comparison | **No direct overlap identified; longitudinal/prognostic task** |
| **Heo et al. (33)** | Prediabetes defined using fasting-glucose and/or HbA1c criteria | None | Predictors were dietary/demographic variables rather than glycaemic diagnostic markers | No | Not applicable | **No direct overlap identified** |
| **Lu et al. (34)** | Prediabetes status created using fasting blood sugar and/or HbA1c criteria | None | Selected variables did not include FBS or HbA1c | No | Not applicable | **No direct overlap identified** |
| **Nguyen et al. (35)** | Prediabetes classification derived from KNHANES clinical examination data; detailed diagnostic threshold was not sufficiently specified in the extracted report | None | Reported model inputs did not include FPG, HbA1c or OGTT glucose | No | Not applicable | **No direct glycaemic overlap identified** |
| **Zhang et al. (36)** | Participants first restricted to normoglycaemia by FBG ≤5.6 mmol/L; AGM versus normal subsequently defined using HbA1c threshold | Fasting glucose; triglyceride–glucose index | FBG contributed to cohort eligibility but was not the marker distinguishing AGM from normal within the included population; HbA1c defined the target but was not a predictor | No | Internal-validation AUC 0.806; no otherwise-identical model without FBG reported | **Criterion-related/partial overlap; not direct same-marker incorporation** |
| **Li et al. (37)** | Prediabetes defined using glucose/HbA1c-related diagnostic criteria | None; glucose and HbA1c were explicitly excluded from candidate predictors | Authors specifically excluded outcome-defining glycaemic markers to avoid statistical bias | No | Not applicable | **No overlap; explicit methodological protection against incorporation bias** |
| **Yang et al. (38)** | Prediabetes status defined according to the clinical/biochemical criteria reported in the original study | No FPG or HbA1c retained; urinary glucose was among candidate predictors | No evidence that the same glycaemic measurement defining prediabetes was used as a retained predictor | No | Not estimable | **No confirmed direct overlap identified** |
| **Yesupatham et al. (39)** | Participants classified as Control or Prediabetes according to HbA1c level | HbA1c, FBG and OGTT glucose | HbA1c directly contributed to the outcome label and was simultaneously used as a model input | No | Internal-test AUC 0.9808; no paired otherwise-identical model excluding HbA1c reported | **Direct predictor–reference-standard overlap: High risk of incorporation bias** |
| **Maalouf et al. (40)** | Prediabetes defined using fasting screening glucose | HbA1c included in some candidate panels; final inflammatory-only model contained no glycaemic marker | HbA1c is glycaemically related but is not the same measurement as the fasting-glucose reference standard; final selected model avoided direct overlap | Partially; alternative feature panels were reported | all_features (including HbA1c and age): AUC 0.734; all_biomarkers (excluding HbA1c and age): AUC 0.734; ΔAUC=0.000. Final inflammatory-only model: AUC 0.743. Difference is not an isolated HbA1c ablation | **No direct same-marker incorporation in the final selected model** |
| **Wang et al. (41)** | I-IGT defined by FPG <6.1 mmol/L with 2-h PG 7.8–<11.1 mmol/L; controls also required FPG <6.1 mmol/L but 2-h PG <7.8 mmol/L | FPG | FPG formed a common eligibility/definition boundary for both groups, whereas 2-h PG distinguished I-IGT from normal; therefore FPG did not directly construct the positive-vs-negative label | No | FPG alone AUC ≈0.946; highest model AUC ≈0.973; no paired model excluding FPG, so inflation cannot be estimated | **Criterion-related/partial overlap; not direct same-marker outcome incorporation** |
| **Pushpanathan et al. (42)** | Prediabetes defined using fasting-glucose and/or HbA1c clinical criteria | None; retinal fundus images only | Reference-standard glycaemic markers were not model inputs | No | Not applicable | **No direct overlap identified** |
| **Fan et al. (43)** | Three-class labels (Normal, Prediabetes, Diabetes) inherited from the Iraqi diabetes dataset; exact rule used to generate the original prediabetes labels was not re-specified in the modelling study | HbA1c among model inputs and selected features | HbA1c is strongly aligned with the clinical class, but the exact original label-generation procedure was insufficiently documented to confirm same-marker incorporation | No | Prediabetes held-out AUC 0.9913; no paired model excluding HbA1c reported | **High concern for potential incorporation bias because HbA1c was a predictor and reference-standard construction was insufficiently described** |
| **Matboli et al. (44)** | Participants classified into healthy, prediabetes and T2DM-related clinical stages using glycaemic/clinical status | Fasting glucose, postprandial glucose and HbA1c included in biochemical/combined feature sets | Glycaemic variables closely corresponding to clinical-stage assignment were entered as predictors | Molecular-only model provides a non-glycaemic comparator, but feature sets differ in multiple dimensions | Molecular-only AUC 0.9956; biochemical AUC 0.9970; combined AUC 0.9989. Combined vs molecular-only ΔAUC=0.0033, but the difference cannot be attributed specifically to glycaemic overlap | **Substantial predictor–reference-standard overlap: High risk of incorporation bias** |
| **Cha et al. (45)** | ADA-based NGT/prediabetes/T2DM classification using FPG, 2-h PG and HbA1c information | Final integrated model used age, sex, BMI, HDL-C, metabolites and SNPs; no FPG, 2-h PG or HbA1c as direct model predictors | Diagnostic glycaemic markers were not included as prediction variables | No | Not applicable | **No direct glycaemic predictor–reference-standard overlap identified** |

**Abbreviations:** AUC, area under the receiver operating characteristic curve; FBG/FPG, fasting blood/plasma glucose; HbA1c, glycated haemoglobin; IFG, impaired fasting glucose; IGT, impaired glucose tolerance; I-IGT, isolated impaired glucose tolerance; NGT, normal glucose tolerance; OGTT, oral glucose tolerance test; 2-h PG, 2-hour post-load/postprandial plasma glucose; SBP, systolic blood pressure; TG, triglycerides; HDL-C, high-density lipoprotein cholesterol; AGM, abnormal glucose metabolism.

**Notes:** Direct predictor–reference-standard overlap was defined conservatively as use of the same glycaemic measurement both as a model predictor and as a component of the contemporaneous reference standard or class-label definition. Related but non-identical glycaemic variables, variables contributing only to study eligibility, and baseline glycaemic predictors measured before subsequent incident-outcome ascertainment were recorded separately and were not automatically classified as direct incorporation bias. “High risk of incorporation bias” refers specifically to this source of bias and does not necessarily correspond to the overall PROBAST judgement, which may also reflect other methodological concerns. The magnitude of AUC inflation attributable specifically to predictor–reference-standard overlap could not be estimated for most studies because otherwise identical models with and without the overlapping glycaemic predictor were generally not reported.

Table S5. Study-level ML-specific methodological audit, descriptive leakage/optimism status, and implications for the PROBAST Analysis domain

| **Study** | **Validation / evaluation separation** | **Preprocessing and missing-data handling relative to validation** | **Feature selection / engineering relative to validation** | **Class-imbalance handling** | **Hyperparameter optimisation / model selection** | **Nested CV or equivalent separation** | **Descriptive ML-specific leakage / optimism status** | **Effect on the PROBAST Analysis-domain judgement** |
| --- | --- | --- | --- | --- | --- | --- | --- | --- |
| Choi et al. (20) | Random split; 10-fold CV | Not reported in sufficient detail | Multivariable analysis; timing relative to split/CV not reported | NR | NR | No nested CV reported | Unclear due to reporting — no explicit leakage demonstrated; workflow sequencing was incompletely reported. | Low retained — no high-risk ML-specific leakage concern was identified. |
| Wang H et al. (21) | Random development/validation split | NR | Univariate and multivariate analysis; timing relative to validation not fully reported | N/A / NR | Model selection details incompletely reported | No nested CV reported; separate validation sample used | Unclear due to reporting — no explicit leakage identified; predictor-selection timing was incompletely described. | Low retained — no high-risk ML-specific leakage concern was identified. |
| Wang L et al. (22) | 5-fold CV | Incomplete records excluded; timing relative to CV not reported | End-to-end ECG/CNN modelling; no separate manual feature-selection procedure reported | NR | NR | No nested CV reported | Unclear due to reporting — preprocessing and tuning sequence was incompletely reported. | Low retained — no high-risk ML-specific leakage concern was identified. |
| Abbas et al. (23) | Random split; 10-fold CV | Imputation reported; whether confined to training data/folds was not reported | Multivariable logistic-regression-based predictor selection; timing relative to validation not reported | NR | NR | No nested CV reported | Unclear due to reporting — placement of imputation and predictor selection relative to validation could not be fully verified. | Low retained — no explicit leakage was identified; residual uncertainty was insufficient to alter the Analysis-domain judgement. |
| Li et al. (24) | Random split; 10-fold CV | NR | Tongue-image feature extraction and feature fusion; placement relative to validation not sufficiently reported | NR | NR | No nested CV reported | Unclear due to reporting — the feature-extraction/training workflow was not sufficiently detailed to fully exclude leakage. | High retained — ML-specific reporting uncertainty was considered together with other Analysis-domain concerns. |
| Dong et al. (25) | Random split; 5-fold CV | No missing data reported | Multivariable logistic-regression-based selection; timing relative to split/CV not reported | NR | NR | No nested CV reported | Unclear due to reporting — timing of data-driven predictor selection relative to validation was unclear. | High retained — uncertainty in data-driven predictor-selection timing contributed to the Analysis-domain concern. |
| Kushwaha et al. (26) | Random split; 10-fold CV | Imputation reported; timing relative to validation not reported | Predictors primarily selected from literature; no clear data-driven dimensionality-reduction step reported | NR | NR | No nested CV reported | Unclear due to reporting — no clear feature-selection leakage was identified, but imputation placement was incompletely reported. | Low retained — no high-risk ML-specific leakage concern was identified. |
| Gong et al. (27) | Random split | Incomplete records excluded; exact sequencing not reported | Literature review followed by univariate and multivariate analyses; timing relative to holdout evaluation not reported | NR | NR | No nested CV reported | Unclear due to reporting — the exact sequence of predictor selection and evaluation was insufficiently reported. | High retained — incomplete sequencing of predictor selection and evaluation was considered together with other Analysis-domain concerns. |
| Hu et al. (28) | Random development/validation split | NR | LASSO selection reported; timing relative to validation not sufficiently specified | N/A / NR | LASSO/model selection reported; tuning details NR | No nested CV reported | Unclear due to reporting — no explicit leakage was demonstrated, but separation of feature/model selection from validation could not be fully verified. | High retained — incomplete separation of feature/model selection from validation was considered together with other Analysis-domain concerns. |
| Ou et al. (29) | Random split; 10-fold CV | NR | LASSO regression; whether selection was performed within resampling folds was not reported | NR | NR | No nested CV reported | Unclear due to reporting — the timing of LASSO feature selection relative to cross-validation could not be confirmed. | High retained — uncertainty about LASSO selection relative to cross-validation contributed to model-selection optimism concerns. |
| Vîrgolici et al. (30) | Random split | No missing data reported | Predictors based primarily on expert knowledge and literature; no formal data-driven reduction reported | NR | Neural-network tuning details NR | No nested CV reported | Unclear due to reporting — no explicit feature-selection leakage was identified, but tuning and preprocessing sequence was incompletely reported. | High retained — no explicit leakage was confirmed; incomplete tuning/workflow reporting was considered together with other Analysis-domain concerns. |
| Chen et al. (31) | Random split; 5-fold CV | Complete-case/deletion approach; timing relative to split not reported | Univariate and multivariate logistic-regression selection; timing relative to CV not reported | NR | NR | No nested CV reported | Unclear due to reporting — feature-selection placement relative to cross-validation could not be verified. | High retained — unclear feature-selection placement relative to cross-validation contributed to concern regarding optimistic performance. |
| Cui et al. (32) | Random split; 5-fold CV | Imputation reported; whether restricted to training data/folds not reported | Multivariable logistic-regression analysis; timing relative to validation not fully described | NR | NR | No nested CV reported | Unclear due to reporting — imputation and predictor-selection timing was incompletely documented. | Low retained — no high-risk ML-specific leakage concern was identified. |
| Heo et al. (33) | Random split; 5-fold CV | Exclusion approach reported; timing relative to CV not fully described | Feature importance/selection based on Gini coefficient; timing relative to resampling not reported | NR | NR | No nested CV reported | Unclear due to reporting — feature-selection timing relative to resampling could not be fully verified. | Low retained — no explicit leakage was identified; incomplete reporting did not alter the Analysis-domain judgement. |
| Lu et al. (34) | Random split | Deletion approach reported; exact timing relative to split not reported | Literature review, data completeness, and collinearity evaluation; sequencing relative to split incompletely reported | NR | NR | No nested CV reported | Unclear due to reporting — no explicit leakage was identified, but some data-dependent screening details were incompletely reported. | Low retained — no high-risk ML-specific leakage concern was identified. |
| Nguyen et al. (35) | Random split | Deletion of incomplete data reported | mRMR feature selection; whether restricted to training data was not reported | NR | HyperTab/model-selection procedures reported, but tuning/evaluation separation not sufficiently detailed | No nested CV reported | Unclear due to reporting — placement of mRMR feature selection and tuning relative to evaluation could not be confirmed. | Low retained — no explicit leakage was demonstrated; incomplete reporting did not alter the Analysis-domain judgement. |
| Zhang et al. (36) | Random split; 10-fold CV | Deletion approach reported; timing relative to validation NR | LASSO regression; timing relative to CV not sufficiently reported | NR | NR | No nested CV reported | Unclear due to reporting — LASSO placement relative to resampling could not be verified. | Low retained — no high-risk ML-specific leakage concern was identified. |
| Li et al. (37) | Random split; 5-fold CV | Imputation reported; whether restricted to training data/folds not reported | LASSO regression; timing relative to split/CV not sufficiently reported | NR | NR | No nested CV reported | Unclear due to reporting — imputation and LASSO placement relative to validation could not be fully verified. | Low retained — no explicit leakage was identified; residual uncertainty did not alter the Analysis-domain judgement. |
| Yang et al. (38) | Random split | NR | LASSO regression; timing relative to split not sufficiently reported | NR | Hyperparameter/model-selection procedures NR | No nested CV reported | Unclear due to reporting — limited reporting prevented exclusion of potential data reuse during feature/model selection. | High retained — limited reporting prevented exclusion of data reuse and supported the Analysis-domain concern. |
| Yesupatham et al. (39) | 70% training / 15% validation / 15% test; 10-fold CV on training set | Missing values reportedly handled during preprocessing, but method and placement not fully described | Pearson correlation and feature-importance analysis; exact timing relative to split not fully reported | NR | NR | Partial safeguard: CV restricted to training set plus separate validation/test sets | Unclear due to reporting; partial safeguard — separate training/validation/test sets reduced leakage risk, but preprocessing and feature-selection sequencing remained incompletely reported. | High retained — separate evaluation sets reduced leakage risk, but residual workflow uncertainty together with other Analysis-domain concerns supported the High-risk judgement. |
| Maalouf et al. (40) | 80/20 development-holdout split; outer 5-fold/inner 3-fold nested CV; separate internal holdout evaluation | Complete analytic dataset; no final imputation described | VIF-based reduction within training folds; biomarker panel and model selected by nested-CV AUC | NR / not clearly required | Model and feature-panel selection conducted within nested CV | Yes – nested CV plus separate holdout evaluation | No clear leakage identified; strong safeguards — nested cross-validation, training-fold-restricted feature reduction, and separate holdout evaluation reduced leakage and model-selection optimism. | Low retained — explicit safeguards protected against information leakage and model-selection optimism. |
| Wang L et al. (41) | Random 7:3 training/validation split | Listwise deletion reported; timing relative to split not specified | Clinical predictors predefined; SHAP/model-based importance used primarily for interpretation | NR | Hyperparameter-tuning details NR | No nested CV reported; separate validation subset used | No clear leakage identified — no data-driven pre-validation feature selection was identified; a separate validation subset was used, although tuning details were incomplete. | Low retained — no high-risk ML-specific leakage concern was identified. |
| Pushpanathan et al. (42) | Individual-specific 70/15/15 internal split; independent external validation in SP2 and BES | Poor-quality/ungradable images excluded; tabular imputation not applicable | End-to-end ViT/ResNet-50 representation learning; no separate full-dataset feature-selection stage reported | NR | Tuning details not fully reported in extracted information | Strong separation through internal train/validation/test sets plus two external cohorts | No clear leakage identified; strong safeguards — internal train/validation/test separation plus independent external validation reduced concern regarding optimistic internal estimates. | Low retained — strong data separation and independent external validation provided safeguards against optimistic internal evaluation. |
| Fan et al. (43) | Stratified 70/10/20 split plus stratified 5-fold CV | Preprocessing restricted to training data | Multiclass LASSO performed on training data only | SMOTE-Tomek restricted to training data | Exact tuning strategy/nested tuning not fully reported | Partial safeguard: held-out test set and training-restricted preprocessing; nested CV not reported | No clear preprocessing/feature-selection leakage identified — preprocessing, LASSO, and SMOTE-Tomek were restricted to training data; tuning remained incompletely reported. | High retained — explicit leakage safeguards were present, but other PROBAST Analysis-domain concerns remained. |
| Matboli et al. (44) | 70/30 train-test split; stratified 5-fold CV | No missing values reported | RFECV and feature-importance analysis; exact placement of RFECV relative to final holdout evaluation not sufficiently described | SMOTE performed within training folds | Hyperparameter/model-selection separation incompletely reported | No nested CV reported | Unclear due to reporting; partial safeguard — SMOTE was restricted to training folds, but the RFECV/model-selection sequence relative to final evaluation remained unclear. | High retained — training-fold-restricted SMOTE provided partial protection, but unclear RFECV/model-selection placement supported the Analysis-domain concern. |
| Cha et al. (45) | Stratified 5-fold CV | Participants with missing genotype, metabolite, or clinical data excluded | Association/FDR screening for metabolites and GWAS p-value screening/LD pruning performed on the full dataset before predictive modelling | NR | No separate nested model-selection procedure reported | No – feature screening preceded CV using the full dataset | High concern — full-dataset association/FDR screening and GWAS-based predictor screening preceded cross-validation, allowing validation observations to influence predictor selection and potentially inflate performance. | High retained — a clear high ML-specific leakage concern directly supported the High-risk Analysis-domain judgement. |

**Abbreviations: CV**, cross-validation; **FDR**, false discovery rate; **GWAS**, genome-wide association study; **LASSO**, least absolute shrinkage and selection operator; **LD**, linkage disequilibrium; **mRMR**, minimum redundancy maximum relevance; **N/A**, not applicable; **NR**, not reported or not reported in sufficient detail; **RFECV**, recursive feature elimination with cross-validation; **SHAP**, SHapley Additive exPlanations; **SMOTE**, synthetic minority oversampling technique; **VIF**, variance inflation factor.

Notes: This structured ML-specific audit supplemented, rather than replaced, the conventional PROBAST assessment. The audit focused on potential information leakage and optimism arising from the separation of training and evaluation data, preprocessing and missing-data handling, feature engineering or feature selection, class-imbalance procedures, hyperparameter optimisation, model selection, and the use of nested cross-validation or equivalent safeguards. When the original publication did not report the sequence of these procedures in sufficient detail, the corresponding item was recorded as NR or described as incompletely reported rather than assumed to represent inappropriate practice. Incompletely reported ML workflows were not automatically classified as high risk. Clear evidence that preprocessing, feature selection, or model selection used information from the full dataset before validation was considered a high-risk ML-specific concern. ML-specific findings were interpreted together with the original PROBAST signalling questions and used to inform the Analysis-domain judgement; they were not treated as an independent fifth PROBAST risk-of-bias domain. The existing PROBAST Analysis-domain classifications of 14 Low-risk and 12 High-risk studies were retained. To make the leakage findings explicit, the descriptive status column uses the labels “No clear leakage identified”, “Unclear due to reporting”, or “High concern”; these are descriptive summaries of the ML-specific audit and do not constitute a separate PROBAST domain. A clear high ML-specific leakage concern was identified for Cha et al. (45), where full-dataset feature screening preceded cross-validation. For the remaining studies, either no clear leakage was identified or leakage could not be excluded because workflow sequencing was incompletely reported. The final column states explicitly whether and how the ML-specific finding affected the PROBAST Analysis-domain judgement.

Table S6. Sensitivity analysis of studies with confirmed direct or substantial predictor–reference-standard overlap

| **Study** | **Nature of overlap** | **Reported performance** | **Role in sensitivity analysis** |
| --- | --- | --- | --- |
| Vîrgolici et al. (30) | Serum glucose and other ATP III components were used both to construct the output label and as model predictors | AUC not reported; test accuracy = 0.96 | Excluded as confirmed direct overlap; did not affect the study-level AUC sensitivity analysis because no AUC was available |
| Yesupatham et al. (39) | HbA1c was used both to define Control/Prediabetes status and as a model input | Internal-test AUC = 0.9808 | Excluded from the study-level AUC sensitivity analysis |
| Matboli et al. (44) | Fasting glucose, postprandial glucose, and HbA1c were included as predictors while glycaemic/clinical status contributed to class assignment | Molecular-only AUC = 0.9956; combined AUC = 0.9989 | Excluded from the primary study-level AUC sensitivity analysis because of substantial overlap |

**Notes:** Sensitivity analysis: After excluding studies with confirmed direct or substantial predictor–reference-standard overlap, 20 current/prevalent identification studies with available AUC values remained. The study-level internal-validation AUC range changed from 0.49–0.9989 to 0.49–0.9913, and the median decreased from 0.821 to 0.813. These changes did not materially alter the interpretation of the evidence. Fan et al. (43) was not classified as confirmed direct overlap because the original class-label construction was insufficiently documented, although it remained a high-concern study for potential incorporation bias.

**Table S7. Study-level missing-data handling in the included studies**

| **Study** | **Missing-data handling** | **Relation to model development/validation** |
| --- | --- | --- |
| Choi et al. (20) | Not reported. | Not reported in sufficient detail. |
| Wang H et al. (21) | Not reported. | Not reported. |
| Wang L et al. (22) | Incomplete records excluded. | Timing relative to 5-fold CV was not reported. |
| Abbas et al. (23) | Imputation reported. | Whether imputation was confined to training data/folds was not reported. |
| Li et al. (24) | Not reported. | Not reported. |
| Dong et al. (25) | No missing data reported. | Not applicable; no missing data were reported. |
| Kushwaha et al. (26) | Imputation reported. | Timing relative to validation was not reported. |
| Gong et al. (27) | Incomplete records excluded. | Exact sequencing relative to validation was not reported. |
| Hu et al. (28) | Not reported. | Not reported. |
| Ou et al. (29) | Not reported. | Not reported. |
| Vîrgolici et al. (30) | No missing data reported. | Not applicable; no missing data were reported. |
| Chen et al. (31) | Complete-case/deletion approach. | Timing relative to the random split was not reported. |
| Cui et al. (32) | Imputation reported. | Whether imputation was restricted to training data/folds was not reported. |
| Heo et al. (33) | Exclusion approach reported. | Timing relative to 5-fold CV was not fully described. |
| Lu et al. (34) | Deletion approach reported. | Exact timing relative to the random split was not reported. |
| Nguyen et al. (35) | Deletion of incomplete data reported. | Timing relative to the random split was not reported in sufficient detail. |
| Zhang et al. (36) | Deletion approach reported. | Timing relative to validation was not reported. |
| Li et al. (37) | Imputation reported. | Whether imputation was restricted to training data/folds was not reported. |
| Yang et al. (38) | Not reported. | Not reported. |
| Yesupatham et al. (39) | Missing values were reported as handled during preprocessing, but the specific method was not described. | The missing-data method and its placement relative to the training/validation/test split were not fully described. |
| Maalouf et al. (40) | Complete analytic dataset reported; no imputation was described for the final analytic data. | No final imputation procedure was described for the analytic dataset. |
| Wang L et al. (41) | Listwise deletion of observations with any missing value. | Timing relative to the 7:3 training/validation split was not specified. |
| Pushpanathan et al. (42) | Poor-quality/ungradable images were excluded; no tabular imputation was applicable. | No tabular imputation was applicable; timing of image-quality exclusion relative to the internal split was not reported. |
| Fan et al. (43) | No imputation method was clearly reported; exact duplicates were removed and format inconsistencies corrected. | Preprocessing was restricted to training data; a specific missing-data/imputation procedure was not reported. |
| Matboli et al. (44) | No missing values reported. | Not applicable; no missing values were reported. |
| Cha et al. (45) | Participants with missing genotype, metabolite, or clinical data were excluded. | Exact timing of exclusion relative to stratified 5-fold CV was not separately reported. |

Abbreviations: CV, cross-validation; N/A, not applicable; NR, not reported or not reported in sufficient detail.

Notes: Missing-data information was reproduced from the included study reports and the existing study-level extraction/audit in this Supplementary Material. When the original publication did not describe the method or its timing relative to model development and validation in sufficient detail, the information was recorded as not reported or unclear rather than inferred.
