# Supplementary material for: Machine learning models for identifying prediabetes: a systematic review of performance, validation, and implications for primary care
Source: Front Med (Lausanne). 2026 Sep 28;13:1956800. doi: 10.3389/fmed.2026.1956800 (PMC13655083; doi:10.3389/fmed.2026.1956800)
Supplement: Supplementary file 4 [file Table4.docx]

**Supplementary Material 3. Transparency of ChatGPT-assisted tasks during peer-review revision**

This supplementary material provides representative requests/prompts used with ChatGPT during peer-review revision, the information supplied to the tool, the intended purpose of each task, and the corresponding human-verification procedure.

**Table S7. Representative requests/prompts, input materials, intended uses, and human-verification procedures for ChatGPT-assisted tasks during peer-review revision**

| **Task** | **Representative request/prompt used** | **Information provided to ChatGPT** | **Intended use** | **Human verification** |
| --- | --- | --- | --- | --- |
| English-language editing | “Please revise the following manuscript text for academic English, improving grammar, clarity, and readability while preserving the original scientific meaning, numerical results, and references. Do not introduce new data or unsupported conclusions.” | Manuscript text prepared by the authors | To improve academic English, grammar, clarity, and readability without changing the scientific content | All revised text was reviewed by the authors and checked against the original manuscript content before incorporation. Numerical results, references, and scientific interpretations were independently verified by the authors. |
| Organisation of extracted study information | “Based only on the study information extracted and provided by the authors, please organise the information into a clear and consistent structure for the manuscript or supplementary table. Do not infer or add information that is not present in the provided material.” | Study information previously extracted by the authors | To assist with structuring author-extracted study information and improving consistency of presentation in the manuscript and supplementary tables | The organised information was checked item by item by the authors against the original publications. ChatGPT did not independently extract study data or determine the accuracy of extracted variables. |
| Consistency checking across the manuscript and supplementary materials | “Please check the manuscript and supplementary-table text provided by the authors for inconsistencies in study numbers, terminology, model-performance values, references, and descriptions. Identify possible inconsistencies for author verification rather than making independent corrections to the underlying study data.” | Manuscript text, supplementary-table text, and author-extracted study information | To identify potential inconsistencies in terminology, study numbers, numerical results, references, and descriptions across manuscript sections and supplementary materials | All potential inconsistencies identified by ChatGPT were manually checked by the authors against the original publications and source data. Final corrections were made only after author verification. |
| Drafting responses to reviewer comments | “Based on the reviewer’s comment and the manuscript text provided, please help draft a point-by-point response and suggest where clarification or revision may be needed. Do not invent data, analyses, references, or methodological procedures that were not performed by the authors.” | Reviewer comments and relevant manuscript or supplementary-material text | To assist with organising and drafting responses to reviewers and identifying manuscript sections that might require clarification | Every response and proposed manuscript revision was reviewed, revised where necessary, and approved by the authors. All scientific statements, data, methodological descriptions, and references were independently verified by the authors before submission. |
| Preliminary structuring and synthesis of information from the updated literature review | “Based on the study information extracted and supplied by the authors from the updated literature search, please help organise and summarise the reported population characteristics, predictors, modelling methods, validation approaches, and performance results. Eligibility, data extraction, and risk-of-bias decisions will be made and verified by the authors.” | Study information extracted by the authors from records identified in the updated literature search | To assist with the preliminary organisation and synthesis of author-extracted information from newly identified studies | Database searching, study eligibility decisions, data extraction, and risk-of-bias assessment were performed by the authors. AI-assisted summaries were manually checked against the original publications before any information was incorporated into the review. |

**Note:** The requests/prompts shown above are representative of the prompts used during peer-review revision. The original prompts were entered primarily in Chinese; English translations are presented here for clarity. ChatGPT (OpenAI; GPT-5.6 Sol) was provided with study information previously extracted by the authors and relevant manuscript or supplementary-material text. It did not independently search bibliographic databases, retrieve source literature, determine study eligibility, perform data extraction, conduct PROBAST or other risk-of-bias assessments, or make independent statistical or scientific interpretations. All AI-assisted outputs were manually reviewed by the authors, and all study-level information, methodological decisions, numerical results, references, and scientific interpretations were verified against the original publications before incorporation into the manuscript. The authors retained full responsibility for all final decisions and content.
