# Supplementary material for: Machine learning models for identifying prediabetes: a systematic review of performance, validation, and implications for primary care
Source: Front Med (Lausanne). 2026 Sep 28;13:1956800. doi: 10.3389/fmed.2026.1956800 (PMC13655083; doi:10.3389/fmed.2026.1956800)
Supplement: Supplementary file 5 [file Table5.docx]

**Supplementary Figure S1. Study-level AUC estimates by validation type and overall PROBAST risk-of-bias classification.**

Each point represents one study-level AUC estimate for a primary current/prevalent prediabetes identification study, stratified by development/internal validation and independent external validation. Point colours indicate the overall PROBAST risk-of-bias classification. Horizontal bars represent reported 95% confidence intervals where available; when a 95% confidence interval was not reported in the original study, only the point estimate is shown. The two independent external-cohort AUC estimates from Pushpanathan et al. (42) are displayed separately to illustrate the attenuation from internal evaluation to external validation. The highest estimate, reported by Matboli et al. (44), represents overall multiclass discrimination rather than a binary prediabetes-specific AUC.
